# A New Basal Sauropod Dinosaur from the Middle Jurassic of Niger and the Early Evolution of Sauropoda

**DOI:** 10.1371/journal.pone.0006924

**Published:** 2009-09-16

**Authors:** Kristian Remes, Francisco Ortega, Ignacio Fierro, Ulrich Joger, Ralf Kosma, José Manuel Marín Ferrer, for the Project PALDES, for the Niger Project SNHM, Oumarou Amadou Ide, Abdoulaye Maga

**Affiliations:** 1 Paleontology, Steinmann Institute for Geology, Mineralogy and Paleontology, University of Bonn, Bonn, Germany; 2 Departamento de Física Matemática y de Fluidos, Facultad de Ciencias, UNED, Madrid, Spain; 3 Museo Paleontológico de Elche (MUPE), Elche, Spain; 4 Staatliches Naturhistorisches Museum Braunschweig, Braunschweig, Germany; 5 Université Abdou Moumouni, Institut pour Recherche et Science Humaine (IRSH), Niamey, Niger; Raymond M. Alf Museum of Paleontology, United States of America

## Abstract

**Background:**

The early evolution of sauropod dinosaurs is poorly understood because of a highly incomplete fossil record. New discoveries of Early and Middle Jurassic sauropods have a great potential to lead to a better understanding of early sauropod evolution and to reevaluate the patterns of sauropod diversification.

**Principal Findings:**

A new sauropod from the Middle Jurassic of Niger, *Spinophorosaurus nigerensis* n. gen. et sp., is the most complete basal sauropod currently known. The taxon shares many anatomical characters with Middle Jurassic East Asian sauropods, while it is strongly dissimilar to Lower and Middle Jurassic South American and Indian forms. A possible explanation for this pattern is a separation of Laurasian and South Gondwanan Middle Jurassic sauropod faunas by geographic barriers. Integration of phylogenetic analyses and paleogeographic data reveals congruence between early sauropod evolution and hypotheses about Jurassic paleoclimate and phytogeography.

**Conclusions:**

*Spinophorosaurus* demonstrates that many putatively derived characters of Middle Jurassic East Asian sauropods are plesiomorphic for eusauropods, while South Gondwanan eusauropods may represent a specialized line. The anatomy of *Spinophorosaurus* indicates that key innovations in Jurassic sauropod evolution might have taken place in North Africa, an area close to the equator with summer-wet climate at that time. Jurassic climatic zones and phytogeography possibly controlled early sauropod diversification.

## Introduction

The Sauropoda were the dominant herbivores in Mesozoic terrestrial ecosystems for at least 120 million years during most of the Jurassic and Cretaceous. In concert with their gigantism [1], this success is unsurpassed by any other group of terrestrial tetrapods. Although the number of known sauropod genera has almost doubled over the last decade, the early evolution of this group is still only poorly understood. This is in part because discoveries from the Early and Middle Jurassic are sparse, especially outside Asia. It has been suggested that Early and early Middle Jurassic sauropods had a Pangaea-wide distribution with a relatively low diversity [2], [3], while continental breakup and regional isolation led to the evolution of endemic groups in the late Middle and Late Jurassic [4]–[7]. However, a general vicariance-driven model for the evolution of dinosaurian faunas has been doubted recently (e.g., [8]). The discovery of the most complete basal sauropod currently known in the Middle Jurassic of North Africa sheds light on the early evolution of this important group, and allows hypothesizing about correlations between sauropod evolution and Jurassic climate and phytogeography.

## Results

### Systematic Paleontology

Dinosauria Owen, 1842 [9]

Saurischia Seeley, 1887 [10]

Sauropoda Marsh, 1878 [11]

*Spinophorosaurus nigerensis*, gen. et sp. nov.

urn:lsid:zoobank.org:act:6469BEE5-EA95-4462-A4E5-4B3277411990

urn:lsid:zoobank.org:act:B2272AD2-5FFB-493A-ABC4-8890D5717859

#### Holotype

**Figure 1:**
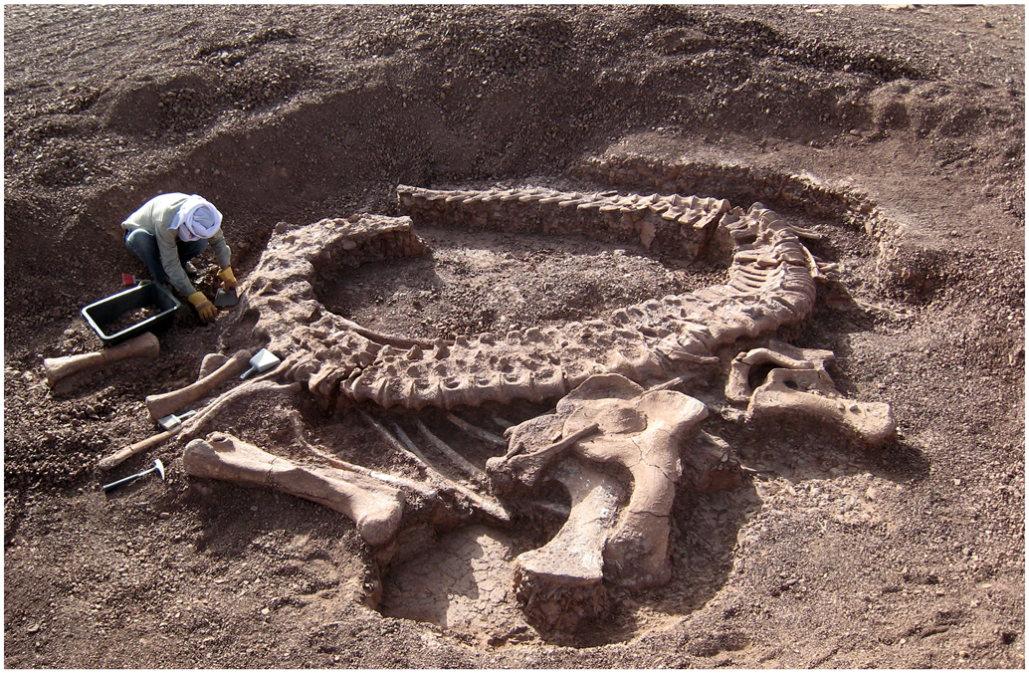
*Spinophorosaurus nigerensis*, holotype skeleton GCP-CV-4229 in situ during excavation in the region of Aderbissinat, Thirozerine Dept., Agadez Region, Republic of Niger.

Specimen numbers GCP-CV-4229 (provisionally housed at the Museo Paleontológico de Elche, Spain; collection abbreviation GCP stands for Grupo Cultural Paleontológico de Elche) and NMB-1699-R (provisionally housed at the Staatliches Naturhistorisches Museum Braunschweig, Germany, collection abbreviation NMB), a braincase, postorbital, squamosal, quadrate, pterygoid, surangular, and a nearly complete postcranial skeleton of a single individual, lacking the sternum, antebrachium, manus, and pes (Fig. 1). In future, the specimens will be managed by the Musée National d'Histoire Naturelle, Niamey, Niger.

#### Paratype

Specimen number NMB-1698-R, a partial skull and incomplete postcranial skeleton. Additional elements not preserved in the holotype individual include the premaxilla, maxilla, lacrimal, dentary, angular (most of these fragmentary), a complete set of right dorsal ribs, the humerus, and an isolated pedal phalanx. The identical morphology of the overlapping elements (postorbital, squamosal, pterygoid, surangular, teeth, axial skeleton, scapula) and the proximity of both skeletons in the same stratigraphical level (see below) justify their referral to the same species.

#### Etymology

The genus name refers to the presence of spike-bearing osteoderms, Latin *spina*, spike, Greek *phoro*, to bear, and *sauros*, lizard. The species epithet refers to the Republic of Niger, the provenance of this taxon.

#### Locality and horizon

The fossils were recovered in an area north of the Rural Community of Aderbissinat (Thirozerine Dept., Agadez Region, Republic of Niger). GPS coordinates may be provided on request; the locality data are archived in the Museo Paleontologico de Elche, Spain and in the Staatliches Naturhistorisches Museum Braunschweig, Germany. The site is located about 30 km to the north and stratigraphically below the outcrops of the Tegama Group in the classic “Falaise de Tiguidit” [12]. Both partial skeletons were found in a massive to finely laminated red siltstone containing some carbonate in its matrix. The siltstone layer is several meters thick and yielded the sauropod remains in its upper half. The holotype and paratype were found in the same level of this layer, about 15 meters laterally apart from each other. In this area, layers are subhorizontal and bear some minor faults. At the top of the unit (about one meter above the level of the skeletons), paleosoils and carbonate deposits are common. Lithostratigraphic characteristics of the area, with units of red clay showing interbedded sand beds (yielding traces of subaerial exposure and some dinosaur footprints), allow the localization of the site at the base of the Irhazer Group (“Argiles de l'Irhazer” below the Tiourarén Formation). The Irhazer Group has traditionally been considered Jurassic to “Neocomian” in age [12]. Subsequently, a Lower Cretaceous age for the Tiourarén Formation has been proposed, carrying important evolutionary and biogeographic implications [13], [14], [8]. Recently, the pre-Aptian and post-Triassic age of the Tiourarén Formation has been critically discussed [15], leading to the conclusion that the represented fauna fits more parsimoniously in a late Middle Jurassic to early Late Jurassic scene [16]. The stratigraphical and phylogenetic position of *Spinophorosaurus* is consistent with an even earlier age, presumably Middle Jurassic (Bajocian-Bathonian). However, since it is currently not possible to date the strata of the Irhazer Group directly, it cannot be excluded that the Argiles de l'Irhazer are even older (Lower Jurassic). The lower age limit is given only by the Teloua sandstones of the underlying Agadez Group, which contain *Chirotherium* trace fossils and are therefore regarded as Upper Triassic [15].

#### Diagnosis

A basal sauropod diagnosed by the following combination of characters (autapomorphies are marked by *): A small pineal foramen that opens dorsally between the contralateral frontals, not parietals*; laterally oriented basal tubera*; spatulate teeth with large, spaced denticles in the apical region, with a higher number of denticles mesially; cranial cervical vertebrae with accessory cranial processes on the prezygapophyses; cervical vertebrae with U-shaped recess between centrum and interpostzygapophyseal lamina (tpol) in lateral view*; triangular caudal process on caudal cervical diapophyses; enlarged cervical epipophyses, having the form of caudally directed, triangular processes; spinodiapophyseal laminae (spdl) restricted to sacral vertebrae; strong rugosities on neural spines extending over the proximal and middle caudal vertebrae; apex of proximal and middle caudal neural spines saddle-shaped*; distal chevrons transformed into overlapping rod-like horizontal elements whose cranial and caudal projections contact at the level of the middle part of the vertebral centra*; kidney-shaped coracoid*; coracoid with large biceps tubercle and furrow on its ventromedial edge; short, robust pubis with an ischial ridge that extends down to the pubic foot; femur shaft with large foramen on its caudal side, lateral to the fourth trochanter*; possession of spike-bearing osteoderms, probably placed in the distal tail region.

### Description and Comparison

Unfused endocranial and neurocentral sutures indicate that the holotype specimen is subadult (vertebral column length ≈13 m; see Table 1 for measurements). The second specimen (NMB-1968-R, about 13% larger; Table 2) has fully fused neurocentral sutures throughout the entire vertebral column.

**Table 1:** Measurements of the holotype individual of *Spinophorosaurus nigerensis*.

Element	Collection number	Distance	Length [mm]
Braincase	GCP-CV-4229	Width	235
Axis	NMB-1699-R	Centrum length	170
3^rd^ cervical vertebra	NMB-1699-R	Centrum length	195
4^th^ cervical vertebra	GCP-CV-4229 (HB 1)	Centrum length	290
12^th^ dorsal vertebra	GCP-CV-4229 (HB 22)	Centrum length	150
		Total height	670
Proximal caudal vertebra	GCP-CV-4229 (HB 31)	Centrum length	110
Middle caudal vertebra	GCP-CV-4229 (HB 44)	Centrum length	146
Distal caudal vertebra	GCP-CV-4229 (HB 101-1)	Centrum length	156
		Total length	280
Left coracoid	GCP-CV-4229 (HB 64′)	Length	525
		Width	310
Left pubis	GCP-CV-4229 (HB 65A+B)	Length	780
Left ischium	NMB-1699-R (1.10)	Length	890
Left femur	GCP-CV-4229 (HB 62)	Length	1215
Left tibia	GCP-CV-4229 (HB 59)	Length	700
Left fibula	GCP-CV-4229 (HB 61)	Length	745
Left astragalus	GCP-CV-4229 (HB 60)	Width	245
		Height	133
Left spike-bearing osteoderm	GCP-CV-4229 (HB 64M)	Total length	290
		Base width	52
		Base length	164

Measurements of selected elements of the holotype individual of *Spinophorosaurus nigerensis*. Most dorsal vertebrae were still in preparation and therefore not accessible for taking measurements at the time of submission of this article.

**Table 2:** Measurements of the paratype individual of *Spinophorosaurus nigerensis*.

Element	Collection number	Distance	Length [mm]
Middle (5^th^?) cervical vertebra	NMB-1698-R (2.35)	Centrum length	320
Middle (6^th^?) cervical vertebra	NMB-1698-R (2.34)	Centrum length	347
Middle (8^th^?) cervical vertebra	NMB-1698-R (2.93)	Centrum length	395
Caudal (10^th^?) cervical vertebra	NMB-1698-R (2.95)	Centrum length	375
Caudal (11^th^?) cervical vertebra	NMB-1698-R (2.99)	Centrum length	395
Middle caudal vertebra	NMB-1698-R (2.47)	Centrum length	140
		Total height	317
Left 3^rd^ thoracic rib	NMB-1698-R (2.R3)	Length	1690
		Maximum shaft width	66
Left scapula	NMB-1698-R (2.37)	Length	1316
		Proximal width	615
		Distal width	355
Left clavicula	NMB-1698-R (2.61)	Length	815
		Maximum width	130
Right humerus	NMB-1698-R (2.38)	Length	1121

Measurements of selected elements of the paratype individual of *Spinophorosaurus nigerensis*.

**Figure 2:**
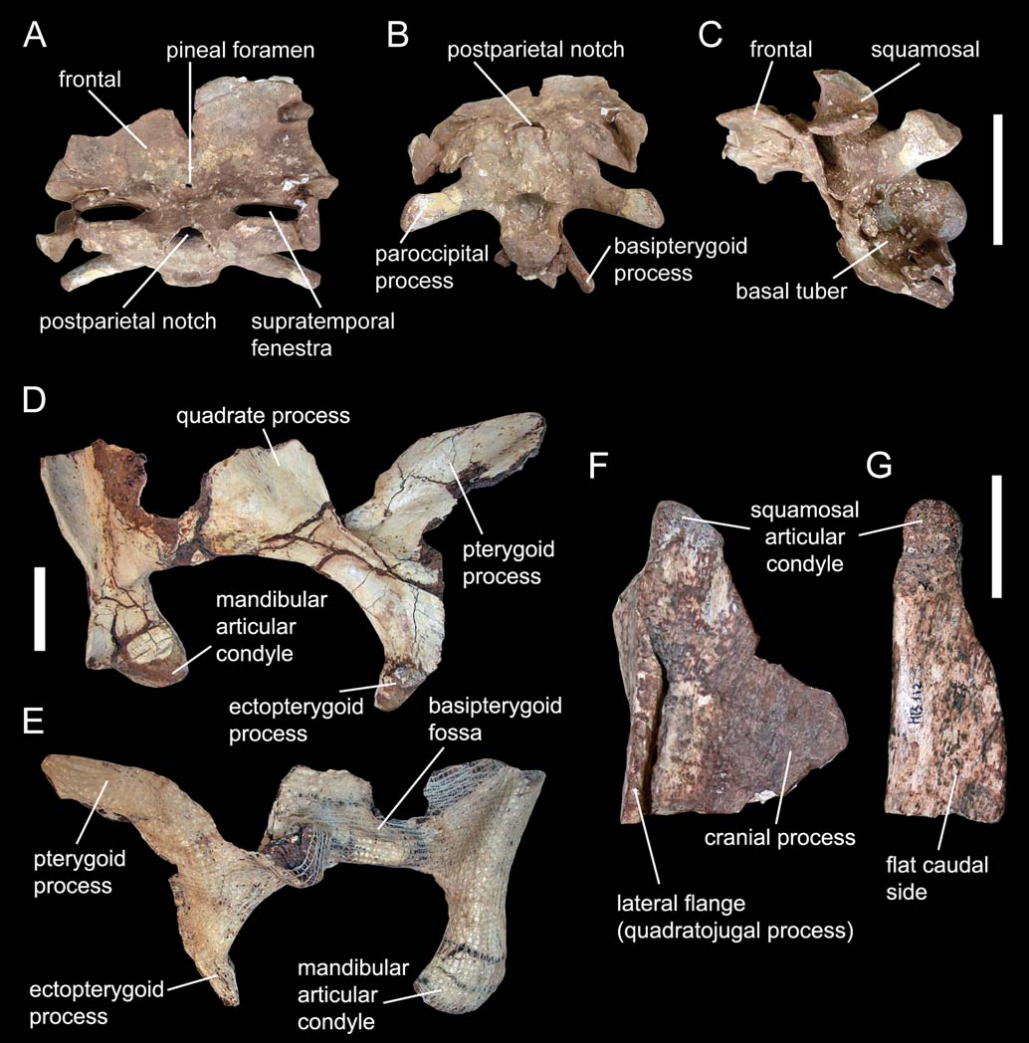
*Spinophorosaurus nigerensis* GCP-CV-4229 (holotype). (A–C)— Braincase in dorsal (A), caudal (B), and left lateral (C) views. (D, E)— Right quadrate and pterygoid in lateral (D) and medial (E) views. (F, G)— Dorsal end of right quadrate in lateral (F) and caudal (G) views. Scale bars  = 10 cm (A–C), 5 cm (D, E), and 2 cm (F, G).

The skull roof of *Spinophorosaurus* (Fig. 2A–C) is characterized by frontals that are, unlike the remaining skull sutures, fused in midline and bear a small median pineal foramen about 10 mm rostral to the frontoparietal suture. The specimen has an open postparietal notch, otherwise known only from dicraeosaurids [16], [17] and the Chinese *Abrosaurus* [18]. The base of the occipital condyle is concave laterally, as in *Shunosaurus* [19]. The enlarged basal tubera are laterally directed, unlike any other known sauropod. The quadrate lacks a concavity on its caudal side (Fig. 2D–G), a plesiomorphic condition otherwise reported only for *Tazoudasaurus* among Sauropoda [20]. The teeth of *Spinophorosaurus* are unique in having spaced, enlarged denticles in the apical region of the crown, with a higher denticle count on the mesial carina (Fig. S1).

**Figure 3:**
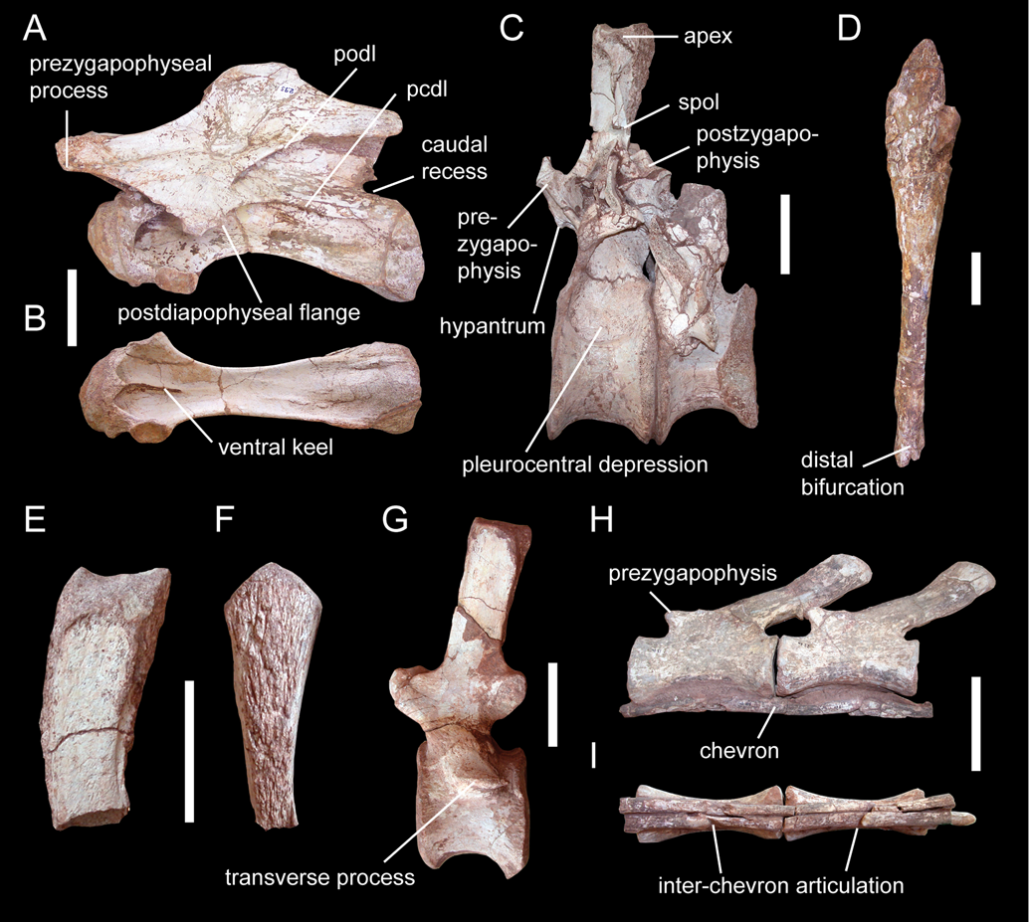
*Spinophorosaurus nigerensis* GCP-CV-4229 (holotype; C, E-I) and NMB-1698-R (paratype; A, B, D). (A, B)— Mid-cervical vertebra in left lateral (A) and ventral (B) views. (C)— Last dorsal and first sacral vertebrae in left lateral view. (D)— Clavicle in cranial view. (E, F)— Proximal caudal neural spines in lateral (E) and cranial (F) views. (G)— Mid-caudal vertebra in lateral view. (H, I)— Distal caudal vertebrae in left lateral (H) and ventral (I) views. Abbreviations: pcdl, posterior centrodiapophyseal lamina; podl, postzygodiapophyseal lamina; spol, spinopostzygapophyseal lamina. Scale bars  = 10 cm.

*Spinophorosaurus* has 25 presacral vertebrae including 13 moderately elongate (elongation index, centrum length without condyle divided by caudal centrum width [21] ≈3.1) cervicals, 4 sacral vertebrae, and more than 37 caudal vertebrae. Cervical centra have large pleurocentral depressions that are deepest cranially, and prominent median crests cranially on their ventral sides (Fig. 3A, B). As in other basal sauropods but different from *Jobaria*, there is no oblique lamina dividing the pleurocoels, nor is the depression on the dorsal side of the parapophysis separated from the remaining pleurocoel [14], [22]–[24]. Cervical neural arches bear triangular accessory processes on the cranial prezygapophyses, which are also known from *Jobaria* but are distinctly deeper in the latter taxon [14]. Moreover, the cervicals have large epipophyses and prominent triangular flanges on the caudal edges of the diapophyses, as known from Middle Jurassic Chinese sauropods [22], [25], [26]. In middle and caudal cervical vertebrae, the base of the neural arch on the centrum shows a U-shaped recess in lateral view. Unlike *Jobaria* [14], there is no deep fossa between the centropostzygapophyseal and interpostzygapophyseal laminae. In general, the cervicals are similar also to the European Middle Jurassic sauropod *Cetiosaurus*, which albeit lacks a ventral keel on the centrum, and has only weak caudal flanges on the cervical diapophyses [24]. When compared to late Early and Middle Jurassic South Gondwanan sauropods, more differences become apparent: The pleurocentral depression is very weak in *Amygdalodon* [27], *Barapasaurus* [28], and *Kotasaurus* [29], [30], but a distinct pleurocoel is present in *Patagosaurus* [31]. The cervical centra generally have lower elongation index values in all forms. In *Barapasaurus*, the lamination of the cervical neural arch is strongly reduced, only a postzygapodiapophyseal lamina can be discerned. The ventral sides of the cervical centra lack a median keel in most South Gondwanan taxa but *Amygdalodon* [27]. Cervical diapophyses are laterally directed (not ventrolaterally) in these forms and lack a caudal flange. In contrast to *Spinophorosaurus* and other northern forms, cervical neural spines have no rugose cranial and caudal faces, and are craniocaudally short and dorsoventrally high in the caudal region of the neck.

While the cranial dorsals bear a distinct pleurocoel, the caudal dorsal centra are short relative to their height in *Spinophorosaurus* and have only a weak pleurocentral depression craniodorsally (Fig. 3C). Dorsal neural arches are characterized by high but very narrow neural canals, and retain a hyposphene-hypantrum articulation up to the last dorsal. The neural spines lack prespinal-, spinodiapophyseal-, and postspinal laminae, but show strong rugosities cranially and caudally that reach ventral to their bases, resembling other basal sauropods [20], [24], [31]. However, caudal cervical and dorsal neural spines of the South Gondwanan forms *Amygdalodon* [32], *Patagosaurus* [31], and *Barapasaurus* [28] are specialized in being craniocaudally short, transversely wide, and having a rounded apex. Moreover, the caudal dorsal vertebrae of *Amygdalodon* and *Patagosaurus* are more elongate relative to their centrum height and have a distinct pleurocentral depression in the dorsal center of the vertebral body [27], [31].

In *Spinophorosaurus*, rugosities on the neural spines extend to the proximal caudal vertebrae (Fig. 3E, F), which is otherwise known only in *Omeisaurus* [22]. As a consequence, the caudal neural arches lack a prespinal lamina and a circular fossa at the base of the spine, characters diagnostic for *Jobaria* [14]. In the distal section of the tail, the neural spines overlap the cranial half of the succeeding vertebrae (Fig. 3H), similar to East Asian sauropods [22], [23] as well as to *Barapasaurus* (KR, pers. obs.) and *Jobaria* [14]. However, in the latter taxon, the reduced postzygapophyses are placed far more distally than in *Spinophorosaurus*.

The dorsal ribcage shows a clear regionalization into pectoral and lumbar ribs, the former (dorsal ribs 2–5) being transversely flattened, backwardly inclined, and with articular facets for sternal ribs distally, the latter (dorsal ribs 6–11) being markedly more slender, with a subcircular cross-section and an increasingly vertical orientation caudal-wards. Among Sauropoda, such a clear regionalization of the ribcage has been described only in one dicraeosaurid [33]; however, complete ribcages are rarely preserved in other forms. In the tail, the proximal chevrons have the plesiomorphic blade-like shape and a bony bridge dorsal to the haemal canal. They lack the rugose ridge across the distal end of the blade characteristic for *Jobaria* [14]. The distal-most chevrons are transformed into consecutive rod-like elements with cranial and caudal ends in contact (Fig. 3H, I). These paired rod-like chevrons lack any connection between the contralateral elements, and have no offset articular facet for the vertebral bodies. Instead, these elements lie closely attached to the ventrolateral edge of the centrum, forming a ventral bracing against lateral and ventral bending of the distal tail, a unique condition among sauropods.

**Figure 4:**
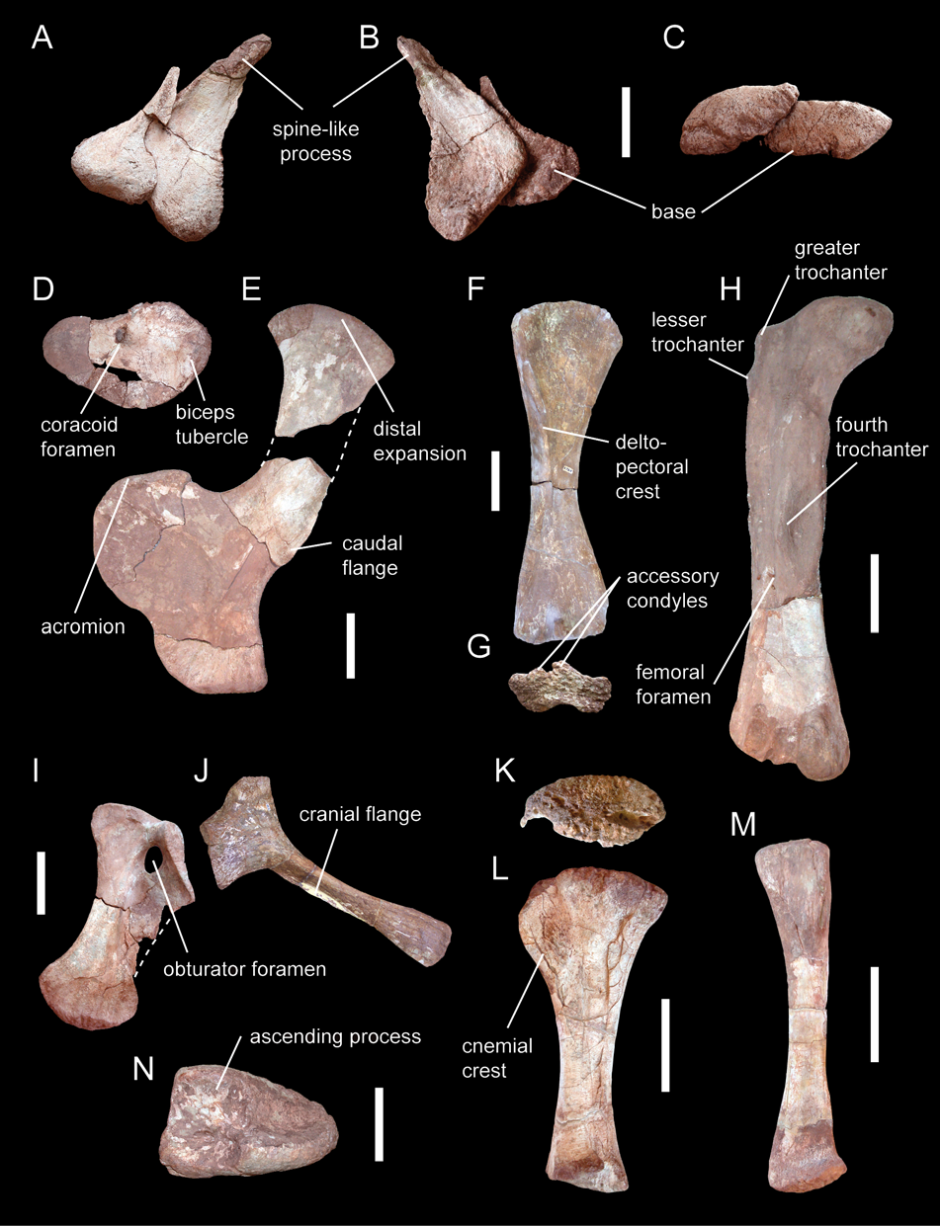
*Spinophorosaurus nigerensis* GCP-CV-4229/NMB-1699-R (holotype; A-E, H-N) and NMB-1698-R (paratype; F, G). (A-C)— Contralateral spike-like osteoderms in dorsolateral (A), ventral (B), and cranial (C) views. (D, E)— Left coracoid (D) and scapula (E) in left lateral views. (F, G)— Right humerus in cranial (F) and distal (G) views. (H)— Left femur in caudal view. (I)— Left pubis in left lateral view. (J)— Left ischium in lateral view. (K, L)— Left tibia in proximal (K) and lateral (L) views. (M)— Left fibula in medial view. (N)— Left astragalus in proximal view. Scale bars  = 10 cm (A–C, N) and 20 cm (D–M).

The scapula is characterized by a strong expansion of the scapular head, a triangular process behind the acromial facet, and a protruding flange on the caudal edge of the blade (Fig. 4E). This character combination is characteristic for mamenchisaurids [34], but an enlarged caudal flange has also been reported in *Cetiosaurus* [35] and *Tehuelchesaurus* [36]. However, in *Vulcanodon*, *Barapasaurus*, and *Patagosaurus* the scapula is straight, only weakly expanded distally, and lacks a distinct caudal flange [28], [31], [37], [38]. The coracoid of *Spinophorosaurus* has a unique kidney-like shape (Fig. 4D). It bears a prominent biceps tubercle and a furrow on its ventromedial edge, characters also found in other basal sauropods [20] (a biceps tubercle is present in coracoids referred to *Kotasaurus*, while a furrow on the ventral edge characterizes the coracoid of *Barapasaurus* [39]). The clavicle is large, robust and has a spear-shaped proximal end (Fig. 3D), but more slender than the clavicle of *Jobaria* [14]. The humerus has a strongly asymmetric distal end with enlarged accessory condyles (Fig. 4F, G), which among other sauropods is known only from mamenchisaurids [22], [23], [34]. The pubis is stout, bearing a caudal flange that connects the pubic foot with the ischial articulation (Fig. 4I). In comparison, the pubic shaft of *Patagosaurus* and *Volkheimeria* is elongate relative to its width and distinctly separate from the proximal part (lacking a connecting caudal flange), with the pubic foot rotated in a transverse orientation [31]. Moreover, *Patagosaurus*, *Barapasaurus*, and *Volkheimeria* are united in having a very slender ischium with only a weak distal expansion [28], [31], while the ischium of other basal sauropods (including *Spinophorosaurus*) is more robust and has a marked distal expansion (Fig. 4J). The femur of *Spinophorosaurus* is characterized by the presence of a lesser trochanter, a large, protruding fourth trochanter with a marked concavity on its medial side, and a unique, large, proximally facing foramen on the shaft lateral to the fourth trochanter (Fig. 4H). On the distal end, the fibular condyle is not markedly smaller than the tibial condyle. The tibia has an oval, craniocaudally elongate proximal end with a craniolaterally directed cnemial crest (Fig. 4K, L), the plesiomorphic condition for sauropods [40], [41]. The fibula is robust with a marked triangular ligament scar proximally (Fig. 4M). The astragalus (Fig. 4N) shows confluent tibial and fibular articular facets without a separating craniocaudal ridge connecting the ascending process and caudal border of the astragalus; moreover, the holotype specimen has an unusually high number of 8 nutritive foramina on its proximal articular surface.

**Figure 5:**
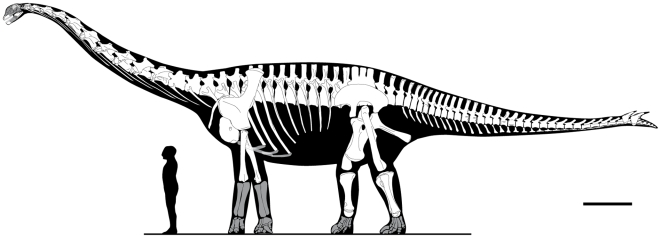
Skeletal reconstruction of *Spinophorosaurus nigerensis*. Dimensions are based on GCP-CV-4229/NMB-1699-R, elements that are not represented are shaded. Scale bar  = 1 m.

With the holotypic skeleton, two closely associated dermal ossifications were found originating from contralateral sides (Fig. 4A–C). These elements have a subcircular base that is rugose and concave on its medial side, and bear a caudodorsally projecting bony spike with a rounded tip laterally. Although these elements were found in the pelvic region under the dislocated scapula, we regard it as most probable that they were placed on the distal tail in the living animal for the following reasons: First, the close association of the contralateral elements indicates they were originally placed near the (dorsal) midline of the body. Second, the stiffening of the distal tail by specialized chevrons is also found in other groups of dinosaurs that exhibit tail armor [42], [43]. Third, osteoderms of similar shape are known from the closely related basal eusauropod *Shunosaurus* [26]. In the latter form, these elements cover the middle part of a tail club formed by coalesced distal vertebrae; however, the decreasing size of the distal-most caudal vertebrae of *Spinophorosaurus* indicate that such a club was not present in this genus. The right osteoderm is slightly larger and differs in proportions from the left element, indicating that, as in *Shunosaurus* [26], originally two pairs of tail spines were present (Fig. 5).

### Phylogenetic Analysis

**Figure 6:**
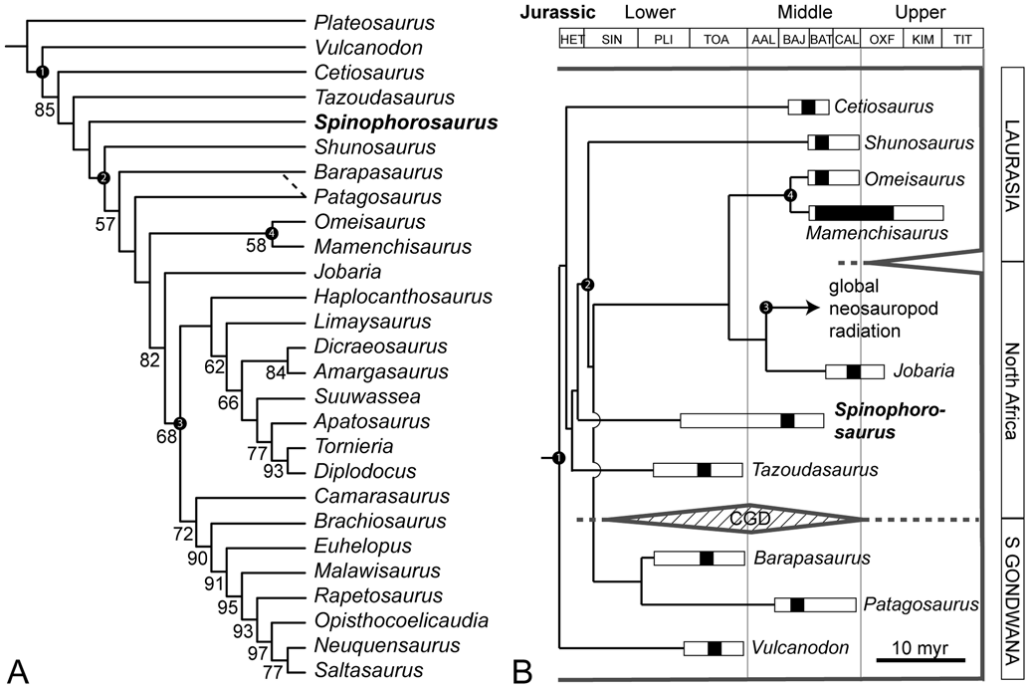
Phylogenetic relationships of *Spinophorosaurus*, based on an analysis of 27 taxa and 235 characters. (A)— Single most parsimonious tree. Numbers next to nodes indicate bootstrap support values for nodes that show more than 50% support. White numbers in black circles: 1, Sauropoda; 2, Eusauropoda; 3, Neosauropoda; 4, Mamenchisauridae. Dashed line indicates alternative sister-group relationship of *Barapasaurus* and *Patagosaurus* as hypothesized in the main text, requiring only a single additional step. (B)— Proposed evolutionary scenario of early sauropods with an endemic South Gondwanan clade during the Lower and Middle Jurassic. White bars indicate insecurities in the dating of the formations in which these taxa were found. Abbreviation: CGD, Central Gondwanan Desert.

A data matrix based on [40] containing 28 operational taxonomical units and 235 anatomical characters (see Methods, Text S1, and Dataset S1) was analyzed with PAUP* 4.0b10 [44], using a heuristic search with random setting and 1000 replicates (following the settings used in [40]). *Plateosaurus* was defined as an outgroup and used to root the tree. The analysis resulted in a single most parsimonious tree (length 515 steps, CI 0.551, RI 0.667, RC 0.368; Dataset S2). Bootstrap analysis was run on the matrix with simple addition heuristic search and 1000 bootstrap replicates. Calculated bootstrap support values of more than 50% are included in Fig. 6A.

Parsimony analysis consistently finds *Spinophorosaurus* to be the sister taxon to Eusauropoda (Fig. 6A), following the original, node-based definition of this clade [41], [45]. The resulting single most parsimonious tree (Dataset S2) was manipulated using MacClade 4.08 [46] to evaluate character evolution and to test several alternative phylogenetic reconstructions. These are (in descending parsimony values): *Barapasaurus*+*Patagosaurus* as sister taxa in a South Gondwanan monophyletic clade (Tree length [TL] increases by 1 step; CI  = 0.55, RC  = 0.37; the same TL increase can be found if a monophyletic clade consisting of *Barapasaurus*+*Patagosaurus* is placed on the tree as sister taxon to Mamenchisauridae, or as sister taxon to *Jobaria*+Neosauropoda); *Spinophorosaurus* as sister taxon of *Tazoudasaurus* (TL increases by 2; CI  = 0.55, RC  = 0.36); *Spinophorosaurus* as sister taxon of *Shunosaurus* (TL increases by 3; CI  = 0.55, RC  = 0.36); *Spinophorosaurus* as sister taxon to *Omeisaurus*+*Mamenchisaurus* (TL increases by 6; CI  = 0.55; RC  = 0.36); *Barapasaurus* and *Patagosaurus* outside *Spinophorosaurus* + Eusauropoda (TL increases by 9, irrespective of whether or not these two taxa form a monophylum; CI  = 0.54, RC  = 0.35); monophyletic Chinese eusauropods (TL increases by 12; CI  = 0.54, RC  = 0.35); and a monophyletic clade of *Spinophorosaurus*, *Shunosaurus* and mamenchisaurids as sister taxon of the remaining Eusauropoda (TL increases by 19; CI  = 0.53; RC  = 0.34). Templeton tests indicate that of these alternatives, only the last three can be rejected by the data with confidence (Table 3).

**Table 3:** Results of Templeton test for various alternative topologies.

Topology	N	n	T_s_	Significance	Comment
AT1	3	1	2	P>0.10	
AT2	3	1	2	P>0.10	
AT3	3	1	2	P>0.10	
AT4	4	1	2.5	P>0.10	
AT5	11	4	24	P>0.10	
AT6	19	7	73.5	P>0.10	
AT7	16	3	19	P<0.05	Significant
AT8	18	2	19	P<0.01	Significant
AT9	25	3	40.5	P<0.005	Significant
AT10	8	3	13.5	P>0.10	
AT11	16	7	59.5	P>0.10	
AT12	16	7	59.5	P>0.10	
AT13	11	5	30	P>0.10	
AT14	16	5	37	P>0.10	

Templeton tests of the significance of tree length differences of various alternative topologies as compared to the most parsimonious tree shown in Fig. 6A. Only alternative topologies AT7, AT8, and AT9 can be rejected by the data with confidence. N  =  number of deviations in step counts found; n  =  number of deviations favoring the alternative topology; T_s_  =  test statistic (summed ranks of n-values). Alternative topologies tested: AT1, *Barapasaurus* + *Patagosaurus* monophyletic sister group to Mamenchisauridae + more derived eusauropods; AT2, *Barapasaurus* + *Patagosaurus* monophyletic sister group to Mamenchisauridae; AT3, *Barapasaurus* + *Patagosaurus* monophyletic sister group to *Jobaria* + more derived eusauropods; AT4, *Spinophorosaurus* + *Tazoudasaurus* monophyletic; AT5, *Spinophorosaurus* + *Shunosaurus* monophyletic; AT6, *Spinophorosaurus* + Mamenchisauridae monophyletic; AT7, *Barapasaurus* + *Patagosaurus* monophyletic sister group to *Spinophorosaurus* + Eusauropoda; AT8, *Shunosaurus* + Mamenchisauridae monophyletic; AT9, *Spinophorosaurus* + *Shunosaurus* + Mamenchisauridae monophyletic sister group to the remaining Eusauropoda; AT10, *Cetiosaurus* sister taxon to *Shunosaurus* + more derived eusauropods; AT11, *Cetiosaurus* sister taxon to *Patagosaurus* + more derived eusauropods; AT12, *Cetiosaurus* sister taxon to *Jobaria* + Neosauropoda; AT13, *Cetiosaurus* sister taxon to *Barapasaurus* + more derived eusauropods; AT14, *Barapasaurus* + *Cetiosaurus* + *Patagosaurus* monophyletic.

The first three alternatives are definitely the most interesting, because they would help to explain the observed differences between North African/Laurasian and South Gondwanan Middle Jurassic sauropods (see Discussion). *Barapasaurus* + more derived eusauropods are united by characters 92, 97, 98, 99, and 107 (see character list contained in the Supporting Information). *Patagosaurus* + more derived eusauropods are supported by characters 72 (presence of presacral pneumatopores, as opposed to mere pleurocentral depressions) and 106 (four sacral vertebrae) only. If *Barapasaurus* + *Patagosaurus* form a monophyletic South Gondwanan clade, the synapomorphies of this clade and more derived eusauropods would include characters 1, 39, 92, 97, 98, 99, 107, and 136 (but not 72 and 106). The autapomorphy of a *Barapasaurus* + *Patagosaurus* clade would be character 101 (presence of subdiapophyseal pneumatopore), which homoplastically is also present in *Cetiosaurus*, *Tazoudasaurus*, and *Mamenchisaurus*. More strikingly, there would be no characters left that unambigously unite Mamenchisauridae + more derived eusauropods.

The very basal position of *Cetiosaurus* within Sauropoda is surprising, and might be due to the incompleteness of this taxon (52.3% of characters unknown). However, low bootstrap support values (Fig. 6A) and tree manipulation data indicate that this position, although being most parsimonious, is not strongly supported. Placing *Cetiosaurus* as sister taxon to *Shunosaurus* + more derived eusauropods, *Patagosaurus* + more derived eusauropods, or *Jobaria* + more derived eusauropods each requires only 2 additional steps; making *Cetiosaurus* the sister taxon to *Barapasaurus* + more derived eusauropods requires only 1 additional step. On the other hand, a monophyletic clade containing *Barapasaurus*, *Patagosaurus*, and *Cetiosaurus* as suggested by some analyses [41] is considerably less parsimonious (5 additional steps). However, Templeton tests show that none of these alternatives can be rejected by the data with confidence (Table 3). Overall, the resolution and node support in this part of the tree remains rather unsatisfactory. Pending ongoing research on Early and Middle Jurassic basal sauropods, it is anticipated that more characters may be included in future analyses that will stabilize the pattern in this part of the tree.

## Discussion

### Phylogenetic position

A strikingly high number of anatomical traits are shared between *Spinophorosaurus* and Middle Jurassic Eurasian forms like *Shunosaurus* and mamenchisaurids. This resemblance is particularly strong among characters of the cervical and caudal vertebrae, the scapula, and the humerus. On the other hand, anatomical differences between *Spinophorosaurus* and Lower and Middle Jurassic South Gondwanan sauropods are numerous (e.g., relative elongation of the cervical vertebrae, development of cervical pleurocoels, shape of cervical and dorsal neural spines, shape of scapula and humerus). Finally, *Spinophorosaurus* shares with *Tazoudasaurus* a number of plesiomorphic traits, such as the lack of a quadrate fossa, and a number of characters of the hind limb.

These observations are meaningful for several reasons. First, the discovery of *Spinophorosaurus* in concert with the new dating for the *Jobaria* sites [15] suggests that these African basal sauropods are distributed chronologically near their phylogenetic relatives in the rest of Gondwana and Asia. Explanations for this novel temporal distribution do not require the previously proposed argument involving slow evolutionary rates [8], [13], [14].

Moreover, earlier analyses [4], [21], [47] suggested that the anatomical peculiarities of Middle Jurassic East Asian sauropods might be the consequence of an endemic radiation. However, more recent analyses [40], [41] contradicted this idea by demonstrating that these taxa do not form a monophyletic clade. In this context, the anatomy and phylogenetic position of *Spinophorosaurus* implies that many of the anatomical traits of East Asian Jurassic sauropods already developed in more basal Sauropoda, and are therefore symplesiomorphic for Eusauropoda. A bootstrap analysis (see above, Fig. 6A) finds that addition of *Spinophorosaurus* and *Cetiosaurus* to a well-established phylogenetic model [40] renders most nodes among basal sauropods unstable, and reduces support for monophyletic Mamenchisauridae. These insecurities can only be resolved by a future detailed analysis of basal sauropod phylogeny, which is beyond the scope of the current work.

On the other hand, the anatomical differences of *Spinophorosaurus* to contemporary South Gondwanan forms contradict the idea of a Pangaean uniformity of sauropod faunas (with the exception of East Asia) prior to the beginning of continental breakup. The explanatory scenario suggested below (see Paleobiogeography), which implies a monophyletic group of South Gondwanan eusauropods, is less parsimonious by only one additional step, and not significantly worse an explanation than the most parsimonious pattern (Table 3). The idea of monophyletic South Gondwanan eusauropods is supported by the fact that a tree containing a monophyletic group consisting of South Gondwanan eusauropods + *Cetiosaurus*, as found by an earlier analysis [41], is considerably less parsimonious, albeit not significantly contradicted by the data (Table 3). Considerably reduced parsimony values can also be found for any other combination of *Barapasaurus*, *Patagosaurus* and North Gondwanan/Laurasian eusauropods.

### Paleobiogeography

Problems with dating the Irhazer Group directly [15], as well as the partial instability of the phylogeny found, render a definite interpretation of the patterns observed difficult. One possible explanation is an origin of the eusauropods in North Gondwana, with the North African *Spinophorosaurus* being closely related to the last common ancestor of all Eusauropoda. In this scenario, the eusauropods that subsequently colonized Laurasia retained characters already acquired by basal forms like *Spinophorosaurus*, while South Gondwanan eusauropods followed a different evolutionary pathway. Given the feasible monophyly of South Gondwanan early sauropods (see above), it might well be that only one lineage of eusauropods invaded South Gondwana in the Lower Jurassic, where it lost many of the characters that unite Laurasian basal sauropods (evolutionary hypothesis proposed in Fig. 6B). However, only additional discoveries of Middle Jurassic sauropods in combination with well-supported phylogenies will reveal if such a model can explain the global distribution of pre-Late Jurassic sauropods better than alternative hypotheses. Nevertheless, from the discovery of *Spinophorosaurus* can be concluded with confidence that there was a connection between North African, European, and East Asian sauropods in the Jurassic.

**Figure 7:**
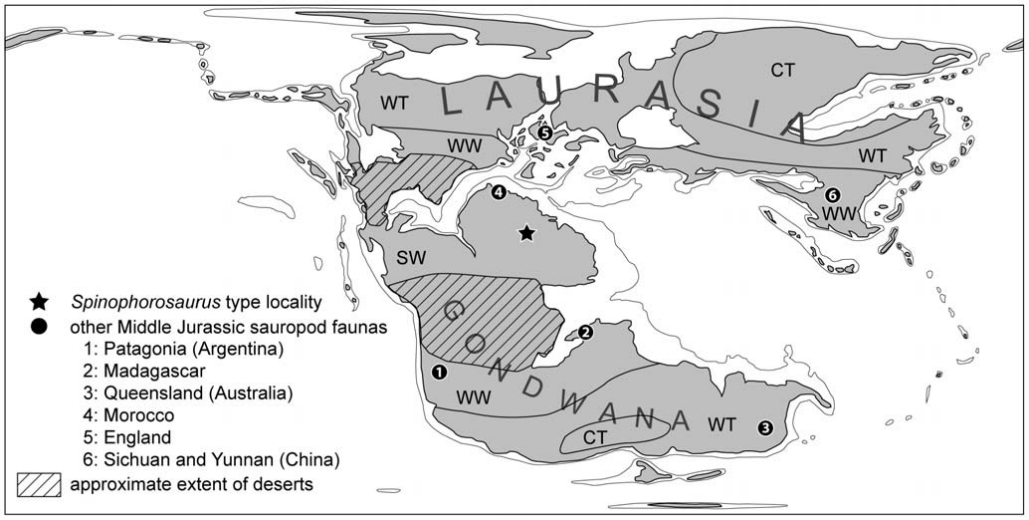
Congruence between Middle Jurassic sauropod distribution and paleoclimatic zones. Although standing close to the origin of Eusauropoda, *Spinophorosaurus* exhibits strong similarities to East Asian Middle Jurassic sauropods (*Shunosaurus*, 6), and much less so to South Gondwanan forms, e.g. the late Lower Jurassic *Barapasaurus* from India and the late Middle Jurassic form *Patagosaurus* (1). The explanation is a separation of global sauropod faunas during the Lower Jurassic by the Central Gondwanan Desert (CGD), forming two different paleobiogeographical domains. Neosauropods had their origin in the same climatic zone as *Spinophorosaurus* and gained global distribution in late Middle Jurassic times (*Atlasaurus*, 4; *Bellusaurus*, 6; *Lapparentosaurus*, 2; *Tehuelchesaurus*, 1). Map redrawn after [48] and [75]. Abbreviations: CT, cold temperate climate; WT, warm temperate climate; SW, summer-wet climate; WW, winter-wet climate.

This previously unrecognized pattern in the distribution of Middle Jurassic sauropod faunas is congruent with paleoclimatic and phytogeographic constraints. A possible correlation between phytogeography, climate, and dinosaur distribution has already been recognized for the Middle and Late Jurassic [48], [49], but may now be traced back to Early Jurassic times at least for the sauropods. In the Early and Middle Jurassic, North Africa was located close to the equator and had a summer-wet climate with high plant productivity and diversity [50], [51]. In contrast, the contemporaneous South Gondwanan and Laurasian sauropod faunas were situated in belts of winter-wet and warm temperate climate (Fig. 7). South Gondwana had been isolated from the equatorial region by the advent of an extensive ecological barrier, the Central Gondwanan Desert (CGD), in the Early Jurassic [15], [51], [52] (Figs. 6B, 7), with immanent differences in the evolution of the North and South Gondwanan floras [48], [53]. After a shrinking of the CGD driven by climate change in late Middle Jurassic times, neosauropods globally replaced typical Middle Jurassic sauropod faunas (Figs. 6B, 7). As indicated by generalized North African forms close to the root of Neosauropoda like *Jobaria* [15], as well as by the wide paleobiogeographic distribution of Upper Jurassic Diplodocoidea [32], [54], [55], the origin of neosauropods and several subgroups may also be located in the Jurassic equatorial region. Following this idea, equatorial Pangaea might be interpreted as a ‘hotspot’ with respect to sauropod evolution, an issue to be explored in more depth in future works. In this context, *Spinophorosaurus* represents a key taxon for understanding the early diversification and ecological specialization of the sauropods in the Jurassic, which obviously was strongly driven also by climatic [49] and phytogeographic factors, and not solely by continental differentiation.

## Methods

### Phylogenetic Analyses

The cladistic analysis was based on [40] and includes the additional modifications of [54]. Moreover, 13 new characters (marked by blue type in the character list, Text S1) were added and scored based on our own observations and published descriptions [14], [16], [19], [20], [22]–[24], [26], [31], [35], [55]–[70].

In addition to *Spinophorosaurus*, codings for *Tazoudasaurus* [20] and *Cetiosaurus* [24], [35], [70] have been added as new OTUs to the original matrix [40], [56]. Moreover, the more incomplete African diplodocid *Tornieria* has been retained from an earlier analysis [55] because of the Gondwanan provenance of this taxon.

On the other hand, a number of incomplete taxa were excluded from the original matrix [40] to increase stability of the tree. These include the rebacchisaurids *Rebacchisaurus* and *Nigersaurus*, which are not relevant to the phylogenetic position of *Spinophorosaurus* since they are widely recognized as representatives of a highly specialized branch of the Diplodocoidea. The same is true for *Barosaurus*, in which the sister-taxon relationship to *Diplodocus* is well established. Furthermore, the incomplete titanosaurs *Nemegtosaurus* and *Alamosaurus* have been excluded from the analysis. As earlier runs of a matrix that included these taxa have shown, none of these deletions influences the phylogenetic position of *Spinophorosaurus*. In addition, the outgroup taxa Theropoda and Prosauropoda were deleted (the latter because of the now widely recognized paraphyletic nature of this clade [71], [72]), and replaced by the single outgroup taxon *Plateosaurus*. The matrix (Dataset S1) also contains *Losillasaurus* (codings from [33]), but this taxon was pruned from the analysis published here because it added a significant degree of instability to higher eusauropods, mainly due to a high percentage of missing data (77.4%).

These modifications led to slight changes in character descriptions, as indicated in the character list (Text S1). Moreover, a number of multistate characters were changed from unordered to ordered. These are characters for which we regard ordered gain or loss of characters as the more plausible assumption than arbitrary character state changes, for example the loss or gain of cervical vertebrae (i.e., the character state change from 15 or more cervicals to 12 cervicals would cost 2 steps in our matrix, while in the original matrix [40] this would have been only a single step). These modifications did not change the topology of the tree, but led to an increase of the support indices of several nodes. Finally, in the original published analyses [40], [56] not applicable characters (as opposed to ‘missing data’) were coded with the symbol ‘9’, which causes problems in the version of MacClade used here since numbers are reserved for character states. One possibility for dealing with inapplicable characters is to code these as gaps. However, gaps (originally designed for the analysis of molecular data) would then either be treated as missing data (and therefore there would be no difference between coding “?” or “−”) or interpreted as a new character state [46]. Therefore, we followed the recommendations in [46], defined inapplicable characters as additional states in the character list, and revised the codings in the data matrix accordingly.

Templeton tests of alternative topologies were conducted following the protocol described in [73], [74] and summarized in [40].

*Spinophorosaurus* was also coded into an alternative published matrix [41]. The analysis resulted in an unsatisfactory polytomy of all basal sauropods below Neosauropoda, which is why we chose [40] as the base for our analysis.

### Nomenclatural Acts

The electronic version of this document does not represent a published work according to the International Code of Zoological Nomenclature (ICZN), and hence the nomenclatural acts contained herein are not available under that Code from the electronic edition. A separate edition of this document was produced by a method that assures numerous identical and durable copies, and those copies were simultaneously obtainable (from the publication date listed on page 1 of this article) for the purpose of providing a public and permanent scientific record, in accordance with Article 8.1 of the Code. The separate print-only edition is available on request from PLoS by sending a request to PLoS ONE, 185 Berry Street, Suite 3100, San Francisco, CA 94107, USA along with a check for $10 (to cover printing and postage) payable to “Public Library of Science”.

The online version of the article is archived and available from the following digital repositories: PubMedCentral (www.pubmedcentral.nih.gov/), and LOCKSS (http://www.lockss.org/lockss/). In addition, this published work and the nomenclatural acts it contains have been registered in ZooBank (http://www.zoobank.org/), the proposed online registration system for the ICZN. The ZooBank LSIDs (Life Science Identifiers) can be resolved and the associated information viewed through any standard web browser by appending the LSID to the prefix “http://zoobank.org/”. The LSID for this publication reads as follows:

urn:lsid:zoobank.org:pub:A704780C-A2F0-47F0-8D66-CFE5A8DE1F91

## Supporting Information

Figure S1 Dentary tooth of *Spinophorosaurus nigerensis*, lingual view (Paratype, NMB-1698-R). Drawing by Ralf Kosma.
(0.63 MB JPG)

Text S1 Character list and character-taxon matrix used in the phylogentic analysis. Includes the character list and the character-taxon matrix used in the phylogenetic analysis, and a list of synapomorphies for relevant nodes with ACCTRAN and DELTRAN modifications.
(0.08 MB DOC)

Dataset S1 NEXUS file of the character-taxon matrix used for the phylogenetic analyses.
(0.03 MB TXT)

Dataset S2 Tree file of most parsimonious tree as found by PAUP for manipulation in MacClade.
(0.00 MB TXT)
