# Supplementary figures and images for: A New Basal Sauropod Dinosaur from the Middle Jurassic of Niger and the Early Evolution of Sauropoda
Source: PLoS One. 2009 Sep 16;4(9):e6924. doi: 10.1371/journal.pone.0006924 (PMC2737122; doi:10.1371/journal.pone.0006924)

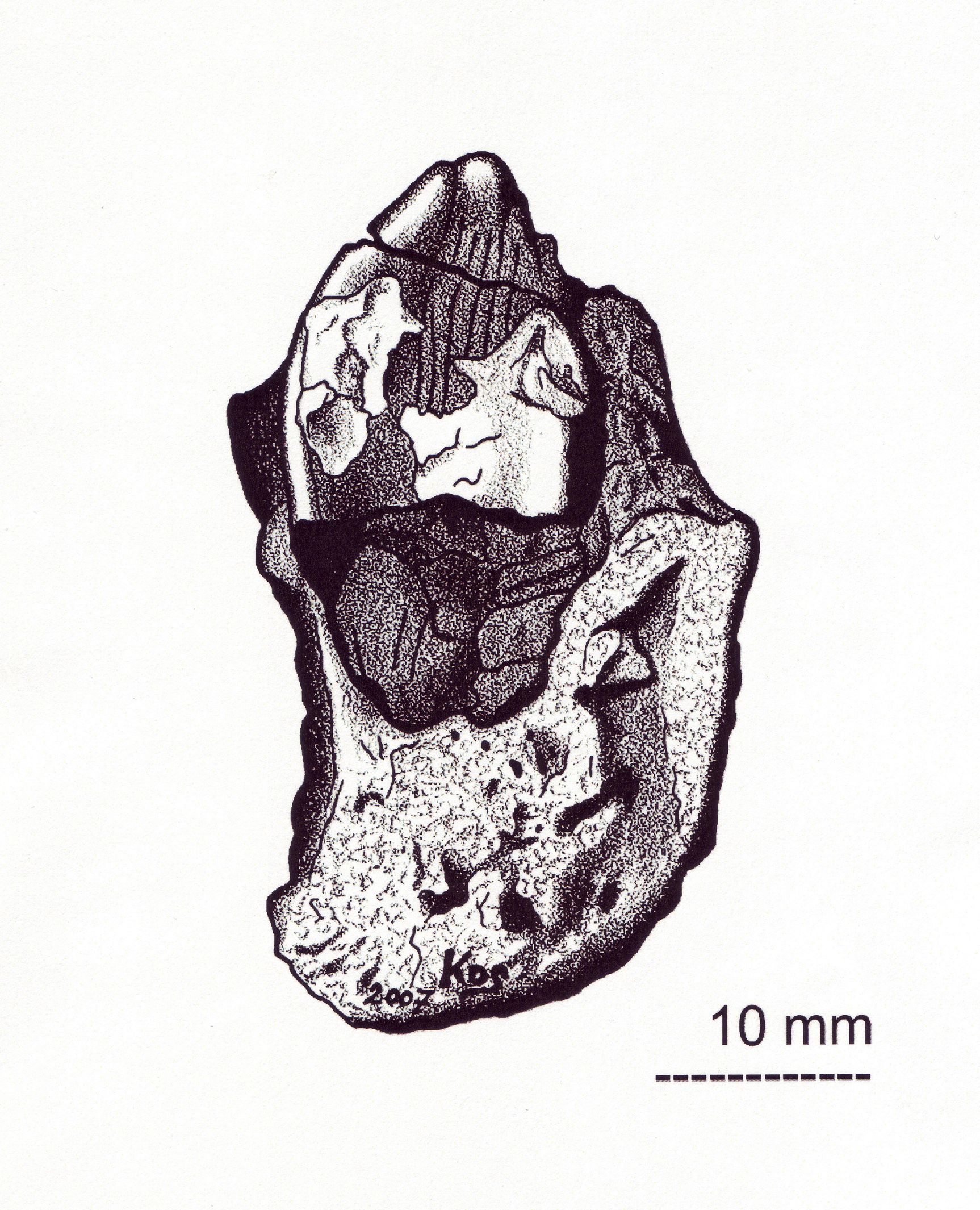

Supplement: Figure S1 — Dentary tooth of Spinophorosaurus nigerensis, lingual view (Paratype, NMB-1698-R). Drawing by Ralf Kosma. (0.63 MB JPG) [file pone.0006924.s001.jpg]
