# Supplementary material for: A New Basal Sauropod Dinosaur from the Middle Jurassic of Niger and the Early Evolution of Sauropoda
Source: PLoS One. 2009 Sep 16;4(9):e6924. doi: 10.1371/journal.pone.0006924 (PMC2737122; doi:10.1371/journal.pone.0006924)
Supplement: Text S1 — Character list and character-taxon matrix used in the phylogentic analysis. Includes the character list and the character-taxon matrix used in the phylogenetic analysis, and a list of synapomorphies for relevant nodes with ACCTRAN and DELTRAN modifications. (0.08 MB DOC) [file pone.0006924.s002.doc]

Character List

The character list is modified from Wilson (2002) (ref. [40], (W001)-(W234); see Methods).

1. (W001) Posterolateral processes of premaxilla and lateral processes of maxilla, shape: without midline contact (0); with midline contact forming marked narial depression, subnarial foramen not visible laterally (1). (Wilson 2002)
2. (W002) Premaxillary anterior margin, shape: without step (0); with marked step, anterior portion of skull sharply demarcated (1). (Wilson 2002)
3. (W003) Maxillary border of external naris, length: short, making up much less than one-fourth narial perimeter (0); long, making up more than one third narial perimeter (1). (Wilson 2002)
4. (W004) Preantorbital fenestra: absent (0); present (1). (Wilson 2002)
5. (W005) Subnarial foramen and anterior maxillary foramen: absent (0); position: well distanced from one another (1); separated by narrow bony isthmus (2). (modified from Wilson 2002)
6. (W006) Antorbital fenestra, maximum diameter: much shorter than (0) or subequal to (1) orbital maximum diameter. (Wilson 2002)
7. (W007) Antorbital fossa: present (0); absent (1). (Wilson 2002)
8. (W008) External nares, position: terminal (0); retracted to level of orbit (1); retracted to a position between orbits (2). [ORDERED] (Wilson 2002)
9. (W009) External nares, maximum diameter: shorter (0) or longer (1) than orbital maximum diameter. (Wilson 2002)
10. (W010) Orbital ventral margin, anteroposterior length: broad, with subcircular orbital margin (0); reduced, with acute orbital margin (1). (Wilson 2002)
11. (W011) Lacrimal, anterior process: present (0); absent (1). (Wilson 2002)
12. (W012) Jugal–ectopterygoid contact: present (0); absent (1). (Wilson 2002)
13. (W013) Jugal, contribution to antorbital fenestra: very reduced or absent (0); large, bordering approximately one-third its perimeter (1). (Wilson 2002)
14. (W014) Prefrontal, posterior process size: small, not projecting far posterior of frontal–nasal suture (0); elongate, approaching parietal (1). (Wilson 2002)
15. (W015) Prefrontal, posterior process shape: flat (0); hooked (1). (Wilson 2002)
16. (W016) Postorbital, ventral process shape: transversely narrow (0); broader transversely than anteroposteriorly (1). (Wilson 2002)
17. (W018) Frontal contribution to supratemporal fossa: no supratemporal fossa (0); present (1); absent (2). (modified from Wilson 2002)
18. (W019) Frontals, midline contact (symphysis): sutured (0) or fused (1) in adult individuals. (Wilson 2002)
19. (W020) Frontal, anteroposterior length: approximately twice (0) or less than (1) minimum transverse breadth. (Wilson 2002)
20. (W021) Parietal occipital process, dorsoventral height: short, less than the diameter of the foramen magnum (0); deep, nearly twice the diameter of the foramen magnum (1). (Wilson 2002)
21. (W022) Parietal, contribution to post-temporal fenestra: present (0); absent (1). (Wilson 2002)
22. (W023) Postparietal foramen: absent (0); present (1). (Wilson 2002)
23. (W024) Parietal, distance separating supratemporal fenestrae: less than (0) or twice (1) the long axis of supratemporal fenestra; supratemporal fenestra reduced (2). (modified from Wilson 2002)
24. (W026) Supratemporal fenestra, long axis orientation: anteroposterior (0); transverse (1); supratemporal fenestra reduced (2). (modified from Wilson 2002)
25. (W027) Supratemporal fenestra, maximum diameter: much longer than (0) or subequal to (1) that of foramen magnum; supratemporal fenestra reduced (2). (modified from Wilson 2002)
26. (W028) Supratemporal region, anteroposterior length: temporal bar longer (0) or shorter (1) anteroposteriorly than transversely; supratemporal fenestra reduced (2). (modified from Wilson 2002)
27. (W029) Supratemporal fossa, lateral exposure: not visible laterally, obscured by temporal bar (0); visible laterally, temporal bar shifted ventrally (1). (Wilson 2002)
28. (W030) Laterotemporal fenestra, anterior extension: posterior to orbit (0); ventral to orbit (1). (Wilson 2002)
29. (W031) Squamosal–quadratojugal contact: present (0); absent (1). (Wilson 2002)
30. (W032) Quadratojugal, anterior process length: short, anterior process shorter than dorsal process (0); long, anterior process more than twice as long as dorsal process (1). (Wilson 2002)
31. (W033) Quadrate fossa: absent (0); present (1). (Wilson 2002)
32. (W034) Quadrate fossa, depth: no quadrate fossa (0), shallow (1); deeply invaginated (2). [ORDERED] (modified from Wilson 2002)
33. (W035) Quadrate fossa, orientation: no quadrate fossa (0); posterior (1); posterolateral (2). [ORDERED] (modified from Wilson 2002)
34. (W036) Palatobasal contact, shape: pterygoid with small facet (0), dorsomedially orientated hook (1), or rocker-like surface (2) for basipterygoid articulation. (Wilson 2002)
35. (W037) Pterygoid, transverse flange (i.e. ectopterygoid process) position: posterior of orbit (0); between orbit and antorbital fenestra (1); anterior to antorbital fenestra (2). [ORDERED] (Wilson 2002)
36. (W039) Pterygoid, palatine ramus shape: straight, at level of dorsal margin of quadrate ramus (0); stepped, raised above level of quadrate ramus (1). (Wilson 2002)
37. (W040) Palatine, lateral ramus shape: plate-shaped (long maxillary contact) (0); rod-shaped (narrow maxillary contact) (1). (Wilson 2002)
38. (W041) Epipterygoid: present (0); absent (1). (Wilson 2002)
39. (W042) Vomer, anterior articulation: maxilla (0); premaxilla (1). (Wilson 2002)
40. (W043) Supraoccipital, height: twice (0) subequal to or less than (1) height of foramen magnum. (Wilson 2002)
41. (W044) Paroccipital process, ventral nonarticular process: absent (0); present (1). (Wilson 2002)
42. (W045) Crista prootica, size: rudimentary (0); expanded laterally into ‘dorsolateral process’ (1). (Wilson 2002)
43. (W046) Basipterygoid processes, length: short, approximately twice (0) or elongate, at least four times (1) basal diameter. (Wilson 2002)
44. (W047) Basipterygoid processes, angle of divergence: approximately 45° (0); less than 30° (1). (Wilson 2002)
45. (W048) Basal tubera, anteroposterior depth: approximately half dorsoventral height (0); sheet-like, 20% dorsoventral height (1). (Wilson 2002)
46. (W049) Basal tubera, breadth: much broader than (0) or narrower than occipital condyle (1). (Wilson 2002)
47. (W050) Basioccipital depression between foramen magnum and basal tubera: absent (0); present (1). (Wilson 2002)
48. (W051) Basisphenoid/basipterygoid recess: present (0); absent (1). (Wilson 2002)
49. (W053) Basipterygoid processes, orientation: perpendicular to (0) or angled approximately 45° to (1) skull roof. (Wilson 2002)
50. Lateral base of occipital condyle flat or slightly convex (0); strongly concave (1).
51. (W054) Occipital region of skull, shape: anteroposteriorly deep, paroccipital processes oriented posterolaterally (0); flat, paroccipital processes oriented transversely (1). (Wilson 2002)
52. (W055) Dentary, depth of anterior end of ramus: slightly less than that of dentary at midlength (0); 150% minimum depth (1). (Wilson 2002)
53. (W056) Dentary, anteroventral margin shape: gently rounded (0); sharply projecting triangular process or ‘chin’ (1). (Wilson 2002)
54. (W058) External mandibular fenestra: present (0); absent (1). (Wilson 2002)
55. (W059) Surangular depth: less than twice (0) or more than two and one-half times (1) maximum depth of the angular. (Wilson 2002)
56. (W060) Surangular ridge separating adductor and articular fossae: absent (0); present (1). (Wilson 2002)
57. (W061) Adductor fossa, medial wall depth: shallow (0); deep, prearticular expanded dorsoventrally (1). (Wilson 2002)
58. (W062) Splenial posterior process, position: overlapping angular (0); separating anterior portions of prearticular and angular (1). (Wilson 2002)
59. (W063) Splenial posterodorsal process: present, approaching margin of adductor chamber (0); absent (1). (Wilson 2002)
60. (W064) Coronoid, size: extending to dorsal margin of jaw (0); reduced, not extending dorsal to splenial (1); absent (2). [ORDERED] (Wilson 2002)
61. (W065) Tooth rows, shape of anterior portions: narrowly arched, anterior portion of tooth rows V-shaped (0); broadly arched, anterior portion of tooth rows U-shaped (1); rectangular, tooth-bearing portion of jaw perpendicular to jaw rami (2). [ORDERED] (modified from Wilson 2002)
62. (W066) Tooth rows, length: extending to orbit (0); restricted anterior to orbit (1); restricted anterior to subnarial foramen (2). [ORDERED] (Wilson 2002)
63. (W067) Crown-to-crown occlusion: absent (0); present (1). (Wilson 2002)
64. (W068) Occlusal pattern: no occlusion (0); interlocking, V-shaped facets (1); high-angled planar facets (2); low-angled planar facets (3). (modified from Wilson 2002)
65. (W069) Tooth crowns, orientation: aligned along jaw axis, crowns do not overlap (0); aligned slightly anterolingually, tooth crowns overlap (1). (Wilson 2002)
66. (W070) Tooth crowns, cross-sectional shape at midcrown: elliptical (0); D-shaped (1); cylindrical (2). (Wilson 2002)
67. (W071) Enamel surface texture: smooth (0); wrinkled (1). (Wilson 2002)
68. (W072) Marginal tooth denticles: present (0); absent on posterior edge (1); absent on both anterior and posterior edges (2). [ORDERED] (modified from Wilson 2002)
69. (W073) Dentary teeth, number: greater than 20 (0); 17 or fewer (1). (Wilson 2002)
70. (W075) Teeth, orientation: perpendicular (0) or oriented anteriorly relative (1) to jaw margin. (Wilson 2002)
71. (W077) Presacral bone texture: solid (0); spongy, with large, open internal cells, ‘camellate’ (Britt, 1993, 1997) (1). (Wilson 2002)
72. (W078) Presacral pneumatopores (pleurocoels): absent (0), present (1). (Wilson 2002)
73. (W079) Atlantal intercentrum, occipital facet shape: rectangular in lateral view, length of dorsal aspect subequal to that of ventral aspect (0); expanded anteroventrally in lateral view, anteroposterior length of dorsal aspect shorter than that of ventral aspect (1). (Wilson 2002)
74. (W080) Cervical vertebrae, number: 10 (0); 12 (1); 13 (2); 15 or greater (3). [ORDERED] (modified from Wilson 2002)
75. (W081) Cervical neural arch lamination: well developed, with well defined laminae and coels (0); rudimentary; diapophyseal laminae only feebly developed if present (1). (Wilson 2002)
76. Cervical vertebrae, median keel on ventral side, absent (0); present (1).
77. (W082) Cervical centra, articular face morphology: amphicoelous (0); opisthocoelous (1). (Wilson 2002)
78. Cervical diapophyses, prominent triangular flange on caudal edge of diapophyseal process, absent (0); present (1).
79. Cervical prezygapophyses, cranial process situated ventrolaterally to articular surface, absent (0); present (1).
80. Cervical epipophyses, absent or rudimentary (0); strongly developed tubercle, projecting caudally (1).
81. (W083) Oblique lamina dividing pleurocoels: absent (0), present (1) (Wilson 2002).
82. (W084) Anterior cervical centra, height:width ratio: less than 1 (0); approximately 1.25 (1). (Wilson 2002)
83. (W085) Anterior cervical neural spines, shape: single (0); bifid (1). (Wilson 2002)
84. (W086) Mid-cervical centra, anteroposterior length/height of posterior face: 2.5–3.0 (0); > 4 (1). (Wilson 2002)
85. (W087) Mid-cervical neural arches, height: less than that of posterior centrum face (0); greater than that of posterior centrum face (1). (Wilson 2002)
86. (W088) Middle and posterior cervical neural arches, centroprezygapophyseal lamina (cprl), shape: single (0); divided (1). (Wilson 2002)
87. (W089) Posterior cervical and anterior dorsal neural spines, shape: single (0); bifid (1). (Wilson 2002)
88. (W090) Posterior cervical and anterior dorsal bifid neural spines, median tubercle: no bifurcation (0); absent (1); present (2). (modified from Wilson 2002)
89. (W091) Dorsal vertebrae, number: 15 (0); 13 (1); 12 (2); 11 (3); 10 or fewer (4). [ORDERED] (modified from Wilson 2002)
90. (W092) Dorsal neural spines, breadth: narrower (0) or much broader (1) transversely than anteroposteriorly. (Wilson 2002)
91. (W093) Dorsal neural spines, length: approximately twice (0) or approximately four times (1) centrum length. (Wilson 2002)
92. (W094) Anterior dorsal centra, articular face shape: amphicoelous (0); opisthocoelous (1). (Wilson 2002)
93. (W095) Middle and posterior dorsal neural arches, centropostzygapophyseal lamina (cpol), shape: single (0); divided (1). (Wilson 2002)
94. (W096) Middle and posterior dorsal neural arches, anterior centroparapophyseal lamina (acpl): absent (0); present (1). (Wilson 2002)
95. (W097) Middle and posterior dorsal neural arches, prezygoparapophyseal lamina (prpl): absent (0); present (1). (Wilson 2002)
96. (W098) Middle and posterior dorsal neural arches, posterior centroparapophyseal lamina (pcpl): absent (0); present (1). (Wilson 2002)
97. (W099) Middle and posterior dorsal neural arches, spinodiapophyseal lamina (spdl): absent (0); present (1). (Wilson 2002)
98. (W100) Middle and posterior dorsal neural arches spinopostzygapophyseal lamina (spol) shape: single (0); divided (1). (Wilson 2002)
99. (W101) Middle and posterior dorsal neural arches, spinodiapophyseal lamina (spdl) and spinopostzygapophyseal lamina (spol) contact: absent (0); present (1). (Wilson 2002)
100. (W102) Middle and posterior dorsal neural spines, shape: tapering or not flaring distally (0); flared distally, with pendant, triangular lateral processes (1). (Wilson 2002)
101. (W103) Middle and posterior dorsal neural arches, ‘infradiapophyseal’ pneumatopore between acdl and pcdl: absent (0); present (1). (Wilson 2002)
102. (W104) Middle and posterior dorsal neural spines, orientation: vertical (0); posterior, neural spine summit approaches level of diapophyses (1). (Wilson 2002)
103. (W105) Posterior dorsal centra, articular face shape: amphicoelous (0); opisthocoelous (1). (Wilson 2002)
104. (W106) Posterior dorsal neural arches, hyposphene–hypantrum articulations: present (0); absent (1). (Wilson 2002)
105. (W107) Posterior dorsal neural spines, shape: rectangular through most of length (0); ‘petal’ shaped, expanding transversely through 75% of ist length and then tapering (1). (Wilson 2002)
106. (W108) Sacral vertebrae, number: 3 or fewer (0); 4 (1); 5 (2); 6 (3). [ORDERED] (modified from Wilson 2002)
107. (W109) Sacrum, sacricostal yoke: absent (0); present (1). (Wilson 2002)
108. (W110) Sacral vertebrae contributing to acetabulum: not applicable (0); numbers 1–3 (1); numbers 2–4 (2); numbers 3–5 (3). (modified from Wilson 2002)
109. (W111) Sacral neural spines, length: approximately twice (0) or four times (1) length of centrum. (Wilson 2002)
110. (W112) Sacral ribs, dorsoventral length: low, not projecting beyond dorsal margin of ilium (0); high extending beyond dorsal margin of ilium (1). (Wilson 2002)
111. (W113) Caudal bone texture: solid (0); spongy, with large internal cells (1). (Wilson 2002)
112. (W115) Caudal transverse processes: persist through caudal 20 or more posteriorly (0); disappear by caudal 15 (1); disappear by caudal 10 (2). [ORDERED] (Wilson 2002)
113. (W116) First caudal centrum, articular face shape: flat (0); procoelous (1); opisthocoelous (2); biconvex (3). (Wilson 2002)
114. (W118) Anterior caudal centra (excluding the first), articular face shape: amphiplatyan or platycoelous (0); procoelous (1); opisthocoelous (2). (Wilson 2002)
115. (W119) Anterior caudal centra, pneumatopores (pleurocoels): absent (0); present (1). (Wilson 2002)
116. (W120) Anterior caudal centra, length: approximately the same (0) or doubling (1) over the first 20 vertebrae. (Wilson 2002)
117. Caudal neural spines, strongly rugose sculpturing extending over entire length of cranial and caudal sides of the spine, absent (0); present (1).
118. (W121) Anterior caudal neural arches, spinoprezygapophyseal lamina (sprl): absent (0); present and extending onto lateral aspect of neural spine (1). (Wilson 2002)
119. (W122) Anterior caudal neural arches, spinoprezygapophyseal lamina (sprl)-spinopostzygapophyseal lamina (spol) contact: absent (0); present, forming a prominent lamina on lateral aspect of neural spine (1). (Wilson 2002)
120. (W123) Anterior caudal neural arches, prespinal lamina (prsl): absent (0); present (1). (Wilson 2002)
121. (W124) Anterior caudal neural arches, postspinal lamina (posl): absent (0); present (1). (Wilson 2002)
122. (W125) Anterior caudal neural arches, postspinal fossa: absent (0); present (1). (Wilson 2002)
123. (W126) Anterior caudal neural spines, transverse breadth: approximately 50% of (0) or greater than (1) anteroposterior length. (Wilson 2002)
124. (W127) Anterior caudal transverse processes, proximal depth: shallow, on centrum only (0); deep, extending from centrum to neural arch (1). (Wilson 2002)
125. (W128) Anterior caudal transverse processes, shape: triangular, tapering distally (0); ‘wing-like’, not tapering distally (1). (Wilson 2002)
126. (W129) Anterior caudal transverse processes, diapophyseal laminae (acdl, pcdl, prdl, podl): absent (0); present (1). (Wilson 2002)
127. (W130) Anterior caudal transverse processes, anterior centrodiapophyseal lamina (acdl), shape: not present (0); single (1); divided (2). [ORDERED] (modified from Wilson 2002)
128. (W131) Anterior and middle caudal centra, shape: cylindrical (0); quadrangular, flat ventrally and laterally (1). (Wilson 2002)
129. (W132) Anterior and middle caudal centra, ventral longitudinal hollow: absent (0); present (1). (Wilson 2002)
130. (W134) Middle and posterior caudal centra, anterior articular face shape: flat (0); procoelous (cone shaped) (1); opisthocoelous (2). (Wilson 2002)
131. (W135) Posterior caudal centra, shape: cylindrical (0); dorsoventrally flattened, breadth at least twice height (1). (Wilson 2002)
132. Posterior caudal spines, orientation: short and dorsally to caudodorsally directed, not extending far beyond the caudal articular facet of the centrum (0); elongate and strongly caudally directed, extending over more than 50% of the length of the succeeding vertebral centrum (1).
133. (W136) Distalmost caudal centra, articular face shape: platycoelous (0); biconvex (1); fused, forming tail club (2). (modified from Wilson 2002)
134. (W137) Distalmost biconvex caudal centra, length-to-height ratio: not applicable (0); less than 4 (1); greater than 5 (2). [ORDERED] (modified from Wilson 2002)
135. (W138) Distalmost biconvex caudal centra, number: none (0); 10 or fewer (1); more than 30 (2). [ORDERED] (modified from Wilson 2002)
136. (W139) Cervical rib, tuberculum–capitulum angle: greater than 90° (0); 90°, rib level with ventral border of centrum (1); less than 90°, rib ventrolateral to centrum (2). [ORDERED] (modified from Wilson 2002)
137. (W140) Cervical ribs, length: much longer than centrum, overlapping as many as three subsequent vertebrae (0); shorter than centrum, little or no overlap (1). (Wilson 2002)
138. (W141) Dorsal ribs, proximal pneumatocoels: absent (0); present (1). (Wilson 2002)
139. (W142) Anterior dorsal ribs, cross-sectional shape: subcircular (0); plank-like, anteroposterior breadth more than three times mediolateral breadth (1). (Wilson 2002)
140. (W143) ‘Forked’ chevrons with anterior and posterior projections: absent (0); present (1). (Wilson 2002)
141. (W144) ‘Forked’ chevrons, distribution: none (0); throughout middle and posterior caudal vertebrae (1); distal tail only (2); reduced to simple U-shaped element (3). [ORDERED] (modified from Wilson 2002)
142. (W145) Chevrons, ‘crus’ bridging dorsal margin of haemal canal: present (0); absent (1). (Wilson 2002)
143. (W146) Chevron haemal canal, depth: short, approximately 25% (0) or long, approximately 50% (1) chevron length. (Wilson 2002)
144. (W147) Chevrons: persisting throughout at least 80% of tail (0); disappearing by caudal 30 (1). (Wilson 2002)
145. (W148) Posterior chevrons, distal contact: fused (0); unfused (open) (1). (Wilson 2002)
146. (W149) Posture: bipedal (0); columnar, obligately quadrupedal posture (1). (Wilson 2002)
147. Scapular head, craniocaudal width, not more than two times the minimum width of the scapular blade (0); strongly expanded, about three times the minimum width of the scapular blade (1). (modified from W150)
148. (W151) Scapular blade, orientation: perpendicular to (0) or forming a 45° angle with (1) coracoid articulation. (Wilson 2002)
149. (W152) Scapular blade, shape: acromial edge not expanded (0); rounded expansion on acromial side (1); racquet-shaped (2). (Wilson 2002)
150. Scapular blade, prominent triangular flange proximally on the caudal edge, absent (0); present (1).
151. (W153) Scapular glenoid, orientation: relatively flat or laterally facing (0); strongly bevelled medially (1). (Wilson 2002)
152. (W154) Scapular blade, cross-sectional shape at base: flat or rectangular (0); D-shaped (1). (Wilson 2002)
153. Scapular head, triangular dorsal process immediately caudal to the acromial articular facet, absent (0); present (1).
154. (W155) Coracoid, proximodistal length: less than (0) or approximately twice (1) length of scapular articulation. (Wilson 2002)
155. (W156) Coracoid, anteroventral margin shape: rounded (0); rectangular (1). (Wilson 2002)
156. (W157) Coracoid, infraglenoid lip: absent (0); present (1). (Wilson 2002)
157. Coracoid, ventromedial edge, convex and rugose (0); with smooth central groove (1).
158. Coracoid, prominent biceps tubercle caudolaterally, present (0); absent (1).
159. (W158) Sternal plate, shape: oval (0); crescentic (1). (Wilson 2002)
160. (W159) Humeral proximolateral corner, shape: rounded (0); square (1). (Wilson 2002)
161. (W160) Humeral deltopectoral attachment, development: prominent (0); reduced to a low crest or ridge (1). (Wilson 2002)
162. (W161) Humeral deltopectoral crest, shape: relatively narrow throughout length (0); markedly expanded distally (1). (Wilson 2002)
163. Humerus, distal articular condyles, subsymmetric (0); strongly asymmetric with enlarged accessory condyles (1).
164. (W163) Humeral distal condyles, articular surface shape: restricted to distal portion of humerus (0); exposed on anterior portion of humeral shaft (1). (Wilson 2002)
165. (W164) Humeral distal condyle, shape: divided (0); flat (1). (Wilson 2002)
166. (W165/166) Ulnar proximal condyle, shape: short lateral process (subtriangular) (0); subequal triradiate (1); unequal triradiate, anterior arm longer (2). [ORDERED] (modified from Wilson 2002)
167. (W167) Ulnar olecranon process, development: prominent, projecting above proximal articulation (0); rudimentary, level with proximal articulation (1). (Wilson 2002)
168. (W168) Ulna, length-to-proximal breadth ratio: gracile (0); stout (1). (Wilson 2002)
169. (W169) Radial distal condyle, shape: round (0); subrectangular, flattened posteriorly and articulating in front of ulna (1). (Wilson 2002)
170. (W170) Radius, distal breadth: slightly larger than (0) or approximately twice (1) midshaft breadth. (Wilson 2002)
171. (W171) Radius, distal condyle orientation: perpendicular to (0) or bevelled approximately 20° proximolaterally (1) relative to long axis of shaft. (Wilson 2002)
172. (W172) Humerus-to-femur ratio: less than 0.60 (0); 0.60 or more (1). (Wilson 2002)
173. (W173) Carpal bones, number: 3 or more (0); 2 or fewer (1); completely lost (2). [ORDERED] (modified from Wilson 2002)
174. (W174) Carpal bones, shape: round (0); block-shaped, with flattened proximal and distal surfaces (1); completely lost (2). (modified from Wilson 2002)
175. (W175) Metacarpus, shape: spreading (0); bound, with subparallel shafts and articular surfaces that extend half their length (1). (Wilson 2002)
176. (W176) Metacarpals, shape of proximal surface in articulation: gently curving, forming a 90° arc (0); U-shaped, forming a 180° arc (1); C-shaped, subtending a 270° arc (2). [ORDERED] (modified from Wilson 2002)
177. (W177) Longest metacarpal-to-radius ratio: close to 0.3 (0); 0.45 or more (1). (Wilson 2002)
178. (W178) Metacarpal I, length: shorter than (0) or longer than (1) metacarpal IV. (Wilson 2002)
179. (W179) Metacarpal I, distal condyle shape: divided (0); undivided (1). (Wilson 2002)
180. (W180) Metacarpal I distal condyle, transverse axis orientation: bevelled approximately 20° proximodistally (0) or perpendicular (1) with respect to axis of shaft. (Wilson 2002)
181. (W181) Manual digits II and III, phalangeal number: 2-3-4-3-2 or more (0); reduced, 2-3-2-2-2 or less (1); absent or unossified (2). [ORDERED] (modified from Wilson 2002)
182. (W182) Manual phalanx I.1, shape: rectangular (0); wedge-shaped (1); lost (2). [ORDERED] (modified from Wilson 2002)
183. (W183) Manual nonungual phalanges, shape: longer proximodistally than broad transversely (0); broader transversely than long proximodistally (1); lost (2). [ORDERED] (modified from Wilson 2002)
184. (W184) Pelvis, anterior breadth: narrow, ilia longer anteroposteriorly than distance separating preacetabular processes (0); broad, distance between preacetabular processes exceeds anteroposterior length of ilia (1). (Wilson 2002)
185. (W185) Ilium, ischial peduncle size: large, prominent (0); low, rounded (1). (Wilson 2002)
186. (W186) Iliac blade dorsal margin, shape: flat (0); semicircular (1). (Wilson 2002)
187. (W187) Iliac preacetabular process, orientation: anterolateral to (0) or perpendicular to (1) body axis. (Wilson 2002)
188. (W188) Iliac preacetabular process, shape: pointed, arching ventrally (0); semicircular, with posteroventral excursion of cartilage cap (1). (Wilson 2002)
189. (W189) Pubis, ambiens process development: small, confluent with (0) or prominent, projecting anteriorly from (1) anterior margin of pubis. (Wilson 2002)
190. (W190) Pubic apron, shape: flat (straight symphysis) (0); canted anteromedially (gentle S-shaped symphysis) (1). (Wilson 2002)
191. (W191) Puboischial contact, length: approximately onethird (0) or one-half (1) total length of pubis. (Wilson 2002)
192. Pubic shaft, elongate and free without continous connection to the puboischial contact (0); shortened and robust, with a caudal flange connecting the distal pubic foot with the puboischial contact (1).
193. (W192) Ischial blade, length: much shorter than (0) or equal to or longer than (1) pubic blade. (Wilson 2002)
194. (W193) Ischial blade, shape: emarginate distal to pubic peduncle (0); no emargination distal to pubic peduncle (1). (Wilson 2002)
195. (W194) Ischial distal shaft, shape: triangular, depth of ischial shaft increases medially (0); bladelike, medial and lateral depths subequal (1). (Wilson 2002)
196. (W195) Ischial distal shafts, cross-sectional shape: Vshaped, forming an angle of nearly 50° with each other (0); flat, nearly coplanar (1). (Wilson 2002)
197. (W196) Femoral fourth trochanter, development: prominent (0); reduced to crest or ridge (1). (Wilson 2002)
198. (W197) Femoral lesser trochanter: present (0); absent (1). (Wilson 2002)
199. (W198) Femoral midshaft, transverse diameter: subequal to (0), 125–150%, or (1) at least 185% (2) anteroposterior diameter. (Wilson 2002)
200. (W199) Femoral shaft, lateral margin shape: straight (0); proximal one-third deflected medially (1). (Wilson 2002)
201. (W200) Femoral distal condyles, relative transverse breadth: subequal (0); tibial much broader than fibular (1). (Wilson 2002)
202. (W201) Femoral distal condyles, orientation: perpendicular or slightly bevelled dorsolaterally (0) or bevelled dorsomedially approximately 10° (1) relative to femoral shaft. (Wilson 2002)
203. (W202) Femoral distal condyles, articular surface shape: restricted to distal portion of femur (0); expanded onto anterior portion of femoral shaft (1). (Wilson 2002)
204. (W203) Tibial proximal condyle, shape: narrow, long axis anteroposterior (0); expanded transversely, condyle subcircular (1). (Wilson 2002)
205. (W204) Tibial cnemial crest, orientation: projecting anteriorly (0) or laterally (1). (Wilson 2002)
206. (W205) Tibia, distal breadth: approximately 125% (0) or more than twice (1) midshaft breadth. (Wilson 2002)
207. (W206) Tibial distal posteroventral process, size: broad transversely, covering posterior fossa of astragalus (0); shortened transversely, posterior fossa of astragalus visible posteriorly (1). (Wilson 2002)
208. (W207) Fibula, proximal tibial scar, development: not well-marked (0); well-marked and deepening anteriorly (1). (Wilson 2002)
209. (W208) Fibula, lateral trochanter: absent (0); present (1). (Wilson 2002)
210. (W209) Fibular distal condyle, size: subequal to shaft (0); expanded transversely, more than twice midshaft breadth (1). (Wilson 2002)
211. (W210) Astragalus, shape: rectangular (0); wedgeshaped, with reduced anteromedial corner (1). (Wilson 2002)
212. (W211) Astragalus, foramina at base of ascending process: present (0); absent (1). (Wilson 2002)
213. (W212) Astragalus, ascending process length: limited to anterior two-thirds of astragalus (0); extending to posterior margin of astragalus (1). (Wilson 2002)
214. (W213) Astragalus, posterior fossa shape: undivided (0); divided by vertical crest (1). (Wilson 2002)
215. (W214) Astragalus, transverse length: 50% more than (0) or subequal to (1) proximodistal height. (Wilson 2002)
216. (W215) Calcaneum: present (0); absent or unossified (1). (Wilson 2002)
217. (W216) Distal tarsals 3 and 4: present (0); absent or unossified (1). (Wilson 2002)
218. (W217) Metatarsus, posture: bound (0); spreading (1). (Wilson 2002)
219. (W218) Metatarsal I proximal condyle, transverse axis orientation: perpendicular to (0) or angled ventromedially approximately 15° to (1) axis of shaft. (Wilson 2002)
220. (W219) Metatarsal I distal condyle, transverse axis orientation: perpendicular to shaft axis (0) or angled dorsomedially to (1) axis of shaft. (modified from Wilson 2002)
221. (W220) Metatarsal I distal condyle, posterolateral projection: absent (0); present (1). (modified from Wilson 2002)
222. (W221) Metatarsal I, minimum shaft width: less than (0) or greater than (1) that of metatarsals II–IV. (Wilson 2002)
223. (W222) Metatarsal I and V proximal condyle, size: smaller than (0) or subequal to (1) those of metatarsals II and IV. (Wilson 2002)
224. (W223) Metatarsal III length: more than 30% (0) or less than 25% (1) that of tibia. (Wilson 2002)
225. (W224) Metatarsals III and IV, minimum transverse shaft diameters: subequal to (0) or less than 65% (1) that of metatarsals I or II (1). (Wilson 2002)
226. (W225) Metatarsal V, length: shorter than (0) or at least 70% (1) length of metatarsal IV. (Wilson 2002)
227. (W226) Pedal nonungual phalanges, shape: longer proximodistally than broad transversely (0); broader transversely than long proximodistally (1). (Wilson 2002)
228. (W227) Pedal digits II–IV, penultimate phalanges, development: subequal in size to more proximal phalanges (0); rudimentary or absent (1). (Wilson 2002)
229. (W228) Pedal unguals, orientation: aligned with (0) or deflected lateral to (1) digit axis. (Wilson 2002)
230. (W229) Pedal digit I ungual, length relative to pedal digit II ungual: subequal (0); 25% larger than that of digit II (1). (Wilson 2002)
231. (W230) Pedal digit I ungual, length: shorter (0) or longer (1) than metatarsal I. (Wilson 2002)
232. (W231) Pedal ungual I, shape: broader transversely than dorsoventrally (0); sickle-shaped, much deeper dorsoventrally than broad transversely (1). (Wilson 2002)
233. (W232) Pedal ungual II–III, shape: broader transversely than dorsoventrally (0); sickle-shaped, much deeper dorsoventrally than broad transversely (1). (Wilson 2002)
234. (W233) Pedal digit IV ungual, development: subequal in size to unguals of pedal digits II and III (0); rudimentary or absent (1). (Wilson 2002)
235. (W234) Osteoderms: absent (0); present (1). (Wilson 2002)

Character-taxon matrix

A nexus file of this matrix is provided as online supporting material.

*Plateosaurus*

0000000000 0000000000 0000000000 0000000000 0000000000 0000000000 0000000000 0000100000 0000000000 0000000000 0000000000 0000000000 0000000000 0000000000 0000000000 0000000000 0000000000 0000000100 0000000000 0000000000 0000000000 0000000000 0000000000 00000

*Vulcanodon*

?????????? ?????????? ?????????? ?????????? ?????????? ?????????? ?????????? ?0???????? 0????????? ?????????? ?????1???? 0?000????? ???100001? ?????????? ?00??1???0 ??0??????? 10?0?21010 01????0??? ????1???00 0010101010 ???000?000 01000010?1 00100100?0 11000

*Tazoudasaurus*

?????????? ?????00?0? ???1????00 00???????? ?????????? ?100010??? 0?1111101? 00??0110?0 01001000?1 ?0011?1010 1000?????? ???0??0??0 0?01000?00 ?000???001 ?00?11?00? ???00000?0 10001?1010 01?101?001 10101?0000 00?0100010 1000011111 0101???00? 011?010001 ?1000

*Barapasaurus*

?????????? ?????????? ?????????? ?????????? ?????????? ?????????? ?????1?0?? 00??001000 0??01000?1 0101101110 100001110? 0?000?0??? ???1000000 01000???01 ?00?01000? 00000011?0 1000121010 0????????? ???0110001 0010101110 1000101111 ?1?10???01 0???????1? ?11?0

*Omeisaurus*

1110001101 10000?1011 00010111?1 1?1?1?1110 0?0?0?0?00 11000?1??? 1111111110 0103011101 11010?0021 01?1101110 0000021201 ?101000000 00010000?0 01???200?1 1000011001 0?10000100 1010111010 0101010000 1110110001 0110101110 00?01011?? 0101001111 1111111111 11110

*Shunosaurus*

0110001101 100000011? ?000011101 1110101100 000000?001 11000?101? 1111111200 0102111100 0100100011 0000?00000 000?010000 0100000000 00010000?0 012000?001 1101010000 0000000100 1000111010 0101010001 1010110001 0010?01110 100?101?10 010?001111 01110111?1 11110

*Patagosaurus*

1?100?11?? ?????????? ?????????? ????????1? ?????????? ??0??????? 1?11111??0 01??011001 1?001000?1 0101101??0 100002??0? ???00?0??? ???1???0?0 00???2?00? ?00??100?0 0?000001?0 1001121010 0????????? ????110001 0010101110 1000001??? ???????101 011??????? ????0

*Spinophorosaurus* n. gen.

?1???????? 1????01111 010101???? 000??0???0 0000011001 1?0?11???? 1??11110?0 01?2011111 0100100021 0?01100000 00000????? 01?0001000 000??00000 0100010001 1000111001 00100010?0 10101????? ?????????? ????1???00 0110101010 0000001111 01000????? ?????????? ????0

*Mamenchisaurus*

1110201110 10000??010 00010?1101 121?1?111? 00????0??0 1100011011 1111111100 1103001010 0101011121 0101101110 1000021?0? ?111001000 00010000?0 01???20001 100?0110?1 ??100?0100 1010121010 011102?000 1???11000? ?010101110 1001101?11 01110?11?1 01?1?1111? ?1??0

*Losillasaurus*

?????????? ?????????? ?????????? ?????????? ??00?????? ?????????? ?????????? ?1??011000 000??100?0 ???1?11011 1?001????? ??110?0000 0?01100?0? ?????????? ?0???????? ????????00 10001???01 ?????????? ??????000? ?????????? ?????????? ?????????? ?????????? ?????

*Cetiosaurus*

?????????? ?????????1 ??010????? ?????????0 00???????? 1????????? ?????????? 01?2001000 0000100021 0100000000 100001???? 0??0000000 0001000000 0????1?0?? ?00??10000 01000?0000 10001210?? ?1???????? ????110001 0010??1110 00001011?0 ?????????? ?????????? ?????

*Suuwassea*

?0???????1 ???????0?? 11110?1??? 111??????? 00?0??10?0 1????????? 2???0?12?? 011?001001 1000?111?? 01???????? ?????????? 0???0????? ?????????0 0?000?10?? ?????10000 01100?01?? 10000????? ?????????? ?????????? ?????????? ???11??11? ?????0?111 110?1?1?10 11???

*Apatosaurus*

0011111201 1?111?1011 1?11011111 11102?1?00 0010000010 1????????? 221302121? 0113001010 1010111241 0111111110 0000021?11 0110000111 1011112000 0012221001 1001010000 1100000100 1000111010 0111120000 1111110011 0010001110 1001101110 1111011111 11111?1111 ?1110

*Tornieria*

11?1??12?? ???01?0??0 1?110????? ?????????0 ???00000?0 1????????? ????0212?? ?1???01??? ???1?????? ?????????? ?????????? 0??111011? ?01?111110 00???????? ?????10000 011?????00 10001210?? ?1???????? ????1100?? ???0001110 1011101010 11110????? ?????????? ?????

*Brachiosaurus*

1111001111 1100011011 0001011111 1210111110 0000001100 1101111112 1111011210 1102001000 1001100021 0101111111 0010021201 0100000001 1001000000 00???2011? ?10??11010 0110000100 1000121010 0111121111 1011110101 1010111111 1001101111 1111001111 0111?11?1? ?11?0

*Camarasaurus*

1111001111 1100011011 0011011100 1211111110 0000000100 1101111111 1111111210 0101001000 1010101121 0101101111 0010021201 0100001001 1011000000 00000200?1 2101011010 0110001100 1000121010 0111121100 1111110001 1110111110 1001101110 1111001111 0111111111 11110

*Dicraeosaurus*

00?11????? 0??00?1111 11111111?? ???1???100 0111010110 111??????? 2213021210 0111011101 1010101221 1111001110 0000121?1? 0110000101 1011100000 0012?21001 ?00??10000 01000001?0 10001210?? ?1???????? ???1110011 0010001110 1001101110 11110??111 111?11???? 11??0

*Diplodocus*

0011111201 1111111011 1011011111 1110211100 0010000110 111101101? 2213021211 0113001000 1011111241 0111111110 0000021211 0111110111 1011112110 0012221001 1001010000 0110000100 1000121010 01???????? ???1110011 0010001110 1011101?10 1111001111 111111111? 11110

*Haplocanthosaurus*

?????????? ?????????? ?????????? ?????????? ?????0???? ?????????? ?????????? 01?2001010 1000100011 0101101111 0000021201 0100001001 1001000000 ?0???2100? ?10??10000 010000?1?? ?????????? ?????????? ???1110001 0010111110 100??????? ?????????? ?????????? ????0

*Amargasaurus*

?????????? 1??0011111 ?111111??? ?????????0 0111011?10 1????????? ?????????? 0012001000 0?10101141 11????1??0 000?12??1? ??0???0??? ?????????0 ?0??????0? ?????10?00 ?10????1?0 10001110?0 01???????? ????1100?? ??????1110 ?00??????? ?????????? ?????????? ????0

*Euhelopus*

11100?11?? 1????1???? ?????????1 111???111? ?????????? ?1011????? 1?11111211 11?3101101 0101101211 0101111?11 011?03??01 ?????????? ?????????? ?????2011? ?????1?00? 10?000???1 10?01????? ?????????? ???1110101 0010111111 1001101?10 111?00?111 01111?1??1 111?0

*Jobaria*

1111001111 1100011011 0001011101 121?1?11?0 00??000?0? 100??????? 1111111000 01?20?1011 0000100021 0101111111 0000021201 010000?001 1001000000 01???20001 ?001010010 010000?100 1000121010 0101120000 1111110001 0?10111110 1001101110 1111001111 011111???? ????0

*Malawisaurus*

11?????1?? 1????????? ?????????? ?????????? ?????????0 ?10??????? 1?1??1121? 11??101010 00011?00?1 0101?01?11 01110????? ?1?10?0001 1101000010 00???201?0 311??1???? ???000?111 1000110010 0???1??111 ?????????? ???111?111 ?????11110 ???????1?? ?????????? ?1??1

*Limaysaurus*

?????????0 1??0012001 102222110? 121??????1 0010101110 1????????? ??1??212?? 01??001?00 10001000?1 ?10???1110 000?1????? 0100000001 1011000000 0012?2???? ?1???10020 0?0000?110 1000121010 01???????? ????11??01 0010111110 ?00?10??1? 1???0??100 01111????? ????0

*Neuquensaurus*

?????????? ?????????? ?????????? ?????????? ?????????? ?????????? ?????????? 11??001000 0?001?00?1 01????1?1? 0111031?0? 1?310?0001 1?11000011 1011?????? ?????10100 100111??11 1101020111 1????????? ????111101 ??01111121 1111111?10 11101??111 0?????1??? ????1

*Opisthocoelicaudia*

?????????? ?????????? ?????????? ?????????? ?????????? ?????????? ?????????? ?1???????? ?????01131 0101111011 0111031?0? ?222000001 1011000012 00111??110 3111110000 1101110111 1101020111 1122121111 2221111?01 1001111121 1101111110 1110111111 0111111110 11110

*Rapetosaurus*

0011?111?1 0?000?0010 101101010? 122211???1 1?0100?10? 10011????? 1112021210 11??101000 0?011?00?1 0101?01011 011103???? ???1??00?? ?????00??1 ?0???2???? ?????10100 ??1100??11 10010?0011 0?????1?1? ???1110101 10011111?1 101??????? ?????????? ?????????? ????1

*Saltasaurus*

?????????? ???00?101? 0011?????? ?????????0 1000101??0 1????????? ?????????? 11??001000 10001100?1 0101?11011 0111031301 1??10?0001 1111000011 1011?2???? ?11?010100 1001110111 1101020111 1????????? ???1111101 1?01111121 1111111110 ?????????? ?????????? ????1

Synapomorphies

In the most parsimonious tree, *Spinophorosaurus* shares the following unequivocal synapomorphies with Eusauropoda (state changes in multistate characters in brackets):

Synapomorphies of *Spinophorosaurus* + Eusauropoda:

Characters 2, 11, 19, 26, 61(1), 112(1), 132, 141(1)

Remaining synapomorphies of Eusauropoda:

Characters 3, 7, 8, 10, 27, 28, 30, 31, 33(1), 35, 37, 38, 57, 59, 62(1), 145, 158, 190, 198, 201, 205, 218, 224, 227, 228, 233, 234

Under ACCTRAN:
Synapomorphies of *Spinophorosaurus* + Eusauropoda:

Characters 19, 30, 57, 61(1), 78, 101, 132, 218, 227, 228, 229, 233, 234

Synapomorphies of Eusauropoda:

Characters 31, 32(1), 33(1), 68(1), 144, 145, 158, 190, 198, 201, 205

Under DELTRAN:

Regarding the clades *Spinophorosaurus* + Eusauropoda and Eusauropoda, no changes of synapomorphic characters come to effect, with the exception of 32(1) as an additional synapomorphy of Eusauropoda (equivocal in the original analysis).
